# Quinazoline-based tricyclic compounds that regulate programmed cell death, induce neuronal differentiation, and are curative in animal models for excitotoxicity and hereditary brain disease

**DOI:** 10.1038/cddiscovery.2015.27

**Published:** 2015-11-30

**Authors:** A Vainshtein, L Veenman, A Shterenberg, S Singh, A Masarwa, B Dutta, B Island, E Tsoglin, E Levin, S Leschiner, I Maniv, L Pe’er, I Otradnov, S Zubedat, S Aga-Mizrachi, A Weizman, A Avital, I Marek, M Gavish

**Affiliations:** 1 Department of Neuroscience, Technion – Israel Institute of Technology, Faculty of Medicine, Rappaport Family Institute for Research in the Medical Sciences, Haifa, Israel; 2 Technion – Israel Institute of Technology, Schulich Faculty of Chemistry, The Mallat Family Laboratory of Organic Chemistry, Haifa, Israel; 3 Department of Physiology, Technion – Israel Institute of Technology, The Behavioral Neuroscience Laboratory, Faculty of Medicine and Emek Medical Center, Haifa, Israel; 4 Tel Aviv University, Sackler Faculty of Medicine, The Felsenstein Medical Research Center, Geha Mental Health Center, Tel Aviv, Israel

## Abstract

Expanding on a quinazoline scaffold, we developed tricyclic compounds with biological activity. These compounds bind to the 18 kDa translocator protein (TSPO) and protect U118MG (glioblastoma cell line of glial origin) cells from glutamate-induced cell death. Fascinating, they can induce neuronal differentiation of PC12 cells (cell line of pheochromocytoma origin with neuronal characteristics) known to display neuronal characteristics, including outgrowth of neurites, tubulin expression, and NeuN (antigen known as ‘neuronal nuclei’, also known as Rbfox3) expression. As part of the neurodifferentiation process, they can amplify cell death induced by glutamate. Interestingly, the compound 2-phenylquinazolin-4-yl dimethylcarbamate (MGV-1) can induce expansive neurite sprouting on its own and also in synergy with nerve growth factor and with glutamate. Glycine is not required, indicating that *N*-methyl-D-aspartate receptors are not involved in this activity. These diverse effects on cells of glial origin and on cells with neuronal characteristics induced in culture by this one compound, MGV-1, as reported in this article, mimic the diverse events that take place during embryonic development of the brain (maintenance of glial integrity, differentiation of progenitor cells to mature neurons, and weeding out of non-differentiating progenitor cells). Such mechanisms are also important for protective, curative, and restorative processes that occur during and after brain injury and brain disease. Indeed, we found in a rat model of systemic kainic acid injection that MGV-1 can prevent seizures, counteract the process of ongoing brain damage, including edema, and restore behavior defects to normal patterns. Furthermore, in the R6-2 (transgenic mouse model for Huntington disease; Strain name: B6CBA-Tg(HDexon1)62Gpb/3J) transgenic mouse model for Huntington disease, derivatives of MGV-1 can increase lifespan by >20% and reduce incidence of abnormal movements. Also *in vitro*, these derivatives were more effective than MGV-1.

## Introduction

The 18 kDa translocator protein (TSPO) takes part in various cellular functions, including regulation of cell death and expression of numerous genes.^1–6^ Associated with these functions the primary locations of the TSPO include mitochondria, and nuclear and perinuclear sites.^1,7,8^ TSPO can be found throughout the body in various tissues.^1,9^ Moderate expression of TSPO also occurs in healthy central nervous system (CNS), which can increase in association with disease and injury, both in glia and in neurons.^10–14^ Programmed cell death and cell differentiation, separately as well as combined, constitute basic, general, and essential functions, for example, regarding embryonic development of the brain, and in adults in response to injury and disease, including cancer.^15–17^ The same is true for gene expression. Since a few years, TSPO function in cell differentiation is also becoming more appreciated.^18^

**Figure 1:**
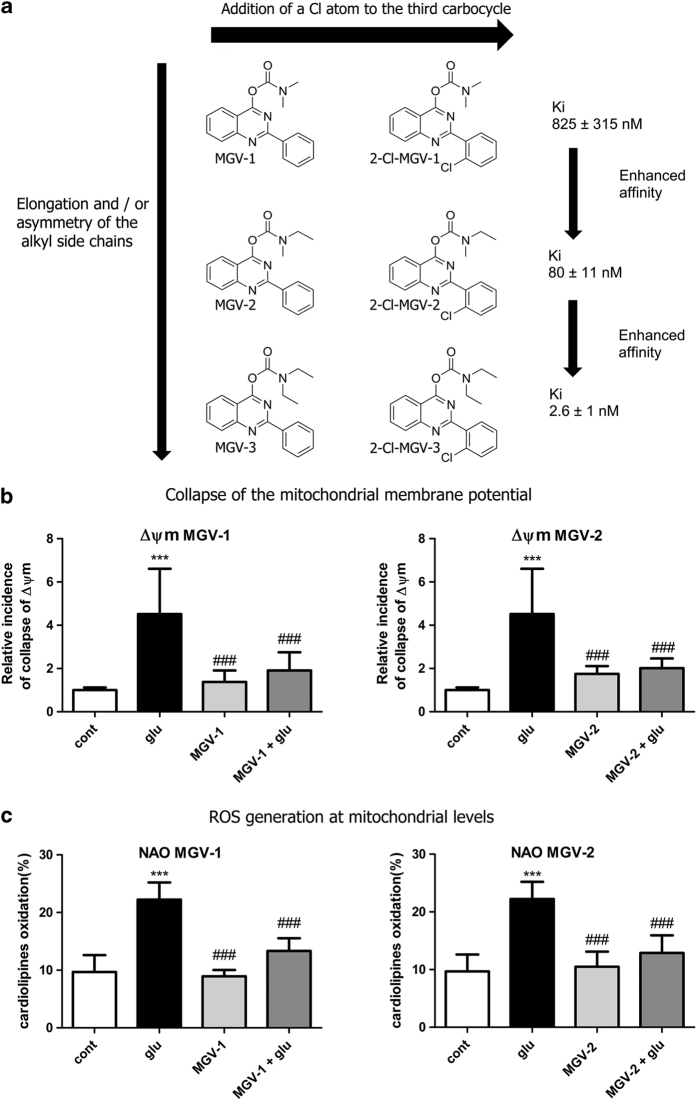
Molecular structures of the tricyclic, quinazoline-based compounds of this study. (**a**) Stepwise changes in the molecular structure from MGV-1 (to 2-Cl-MGV-1, MGV-2, 2-Cl-MGV-2, MGV-3, and 2-Cl-MGV-3, respectively). Note: the stepwise changes include additions of single C atoms to the methyl side chains, and adding a Cl substituent to the third, rotatable carbocycle. Elongation of the methyl chains to ethyl (symmetric as well as asymmetric) leads to enhanced affinity. Addition of a Cl substituent to the second position in the third rotatable carbocycle does not affect affinity. Affinity is presented as Ki derived from displacement of [^3^H]PK 11195. (**b**) These compounds can counteract collapse of the ΔΨm induced by 35 mM of glutamate, for example, by 25 *μ*M of MGV-1 as well as MGV-2, as assayed with JC-1. (**c**) MGV-1 and MGV-2 (25 *μ*M) also prevent of mitochondrial ROS generation induced by glutamate (35 mM), as assayed by NAO. The results are expressed as mean±S.D. ANOVA followed by the Bonferroni correction for multiple comparisons as *post hoc*: ***P<0.001 *versus* control, ^###^*P*<0.001 *versus* glut 35 mM (in **b**, *n*=12; in **c**, *n*=8).

We have developed relatively small (~400  molecular weight) tricyclic compounds, based on the bicyclic quinazoline as a scaffold, to regulate the functions of the TSPO^19,20^ (Figure 1). In the present project, we studied their ability to modulate TSPO functions related to cell death induced by overload of glutamate, first of all in cell culture, and also in animal models (systemic injections of kainic acid in rats and the R6-2 transgenic mouse model for Huntington disease). We also studied the effects of these agents on neurodifferentiation in culture. It has been shown previously that TSPO present in progenitor cells for glia and neurons are associated with their differentiation, presenting TSPO as a promising target for research in this area.^13^

Both glial cells and neuronal cells are major components of embryonic development of the CNS, as well as brain damage related processes because of injury and disease.^21,22^ Therefore, we studied the U118MG (glioblastoma cell line of glial origin) and the PC12 cell line (cell line of pheochromocytoma origin with neuronal characteristics). PC12 cells have long been used as a model for neurodifferentiation, typically induced by application of nerve growth factor (NGF).^23–25^ We applied glutamate to induce cell death in these cell lines. We then compared the effects of the mentioned tricyclic compounds, NGF, and glutamate on cell death and differentiation. In this context, during embryonic CNS development neuronal progenitor cells migrate to their target brain areas.^26^ There they distribute along scaffolds established by radial glia.^27^ Interactions with the glia are thought to contribute to differentiation of progenitor cells to mature neurons.^28^ The vast majority of progenitor cells do not differentiate and are weeded out by programmed cell death.^29–31^ Regarding trauma of healthy mature brain tissue, programmed cell death can result from various mechanic and toxic insults, and disease, including expanding brain cancer.^32,33^ In adults of many species of vertebrates, migration of progenitor cells into damaged brain areas can allow for replenishment of depleted neurons.^34–37^ Regarding brain cancer, glioma cells are almost by definition not differentiated and resistant to programmed cell death.^38^ At the same time, they induce cell death in surrounding healthy tissue, for example, by excessive glutamate release, as well as by mechanic compression of the brain in its enclosure of the skull.^39^

For the present article, we studied whether the effects regarding cell death and differentiation, of our tricyclic compounds based on the bicyclic quinazoline scaffold, are associated with mitochondrial functions, including metabolic rate, maintenance or collapse of the mitochondrial membrane potential (ΔΨm), generation of reactive oxygen species (ROS), cardiolipin peroxidation, all of them functions that are known to be under the control of the TSPO and also modulated by glutamate application.^3,5,40,41^ Regarding neurodifferentiation, we examined and quantified the sprouting of neurites, the hallmark of neurodifferentiation.^23–25^ In addition, we studied expression of *β*-actin, tubulin-3*β*, and NeuN (antigen known as ‘neuronal nuclei’, also known as Rbfox3), as they present molecular biological markers for neurodifferentiation and mature neurons.^42^ We also examined whether TSPO and glial fibrillary acidic protein (GFAP) expression change in our compound-induced neurodifferentiation.

To discern whether our cell culture findings of cell death and cell differentiation may actually translate to implications for the whole organism, we applied our novel TSPO active agents to established mammalian models for progressing brain damage and neurodegeneration. These models included systemic kainic acid injections in rats to induce localized excitotoxic damage in hippocampus, amygdala, and pyriform cortex, and subsequent edemic damage spread throughout the brain.^43,45–48^ We also studied the R6-2 transgenic mouse model for the human hereditary neurodegenerative disease of Huntington, which presents damage in the striatum and cortex, motor disturbances, and finally, early death.^49,50^ Thus, we endeavored to find out whether these tricyclic compounds based on a quinazoline scaffold could counteract the damage and behavioral deficits and potentially even restore the aberrant behavior to normal. And last but not least, whether these compounds can prolong the lifespan of the animals in question.

## Results

### Cell culture studies

The novel tricyclic compounds based on a quinazoline scaffold of this study were tested by us, for example, to attenuate glutamate-induced cell death of the human glioblastoma cell line U118MG and to provide neuroprotective effects in animal models for brain injury and brain disease.^19,20^ Based on results from several generations of drugs designed by us, this study focusses on six compounds: 2-phenylquinazolin-4-yl dimethylcarbamate (MGV-1), 2-(2-chlorophenyl)quinazolin-4-yl dimethylcarbamate (2-Cl-MGV-1), 2-phenylquinazolin-4-yl ethyl(methyl)carbamate (MGV-2), 2-(2-chlorophenyl)quinazolin-4-yl ethyl(methyl)carbamate (2-Cl-MGV-2), 2-phenylquinazolin-4-yl diethylcarbamate (MGV-3), and 2-(2-chlorophenyl)quinazolin-4-yl diethylcarbamate (2-Cl-MGV-3) (Figure 1). In Figure 1a, it can be seen that adding each time one C-atom to the alkyl side chains improves the affinity. Applying displacement studies of these compounds in comparison with the classical TSPO ligand PK 11195 (the classical TSPO ligand: *N*-butan-2-yl-1-(2-chlorophenyl)-*N*-methylisoquinoline-3-carboxamide), the affinities (Ki’s) presented in Figure 1a show that the affinities of MGV-3 and 2-Cl-MGV-3 are similar to PK 11195 (~2.5 nM), whereas those of MGV-2 and 2-Cl-MGV-2 are one order of magnitude lower, and those of MGV-1 and 2-Cl-MGV-1 are two orders of magnitude lower (Figure 1a), as determined in rat kidney homogenate, using [^3^H]PK 11195 as a radiolabeled TSPO ligand. We assumed that our compounds would prevent collapse of the ΔΨm, which is typically induced by glutamate^51^ and normally under the control of the TSPO.^4,5^ Applying JC-1 (5,5′,6,6′-tetrachloro-1,1′,3,3′-tetraethylbenzimidazolylcarbocyanine iodide a cationic carbocyanine dye that accumulates in mitochondria) showed that the cell protective effects of MGV-1 and MGV-2 (25 *μ*M) indeed include prevention of the collapse of the ΔΨm and generation of ROS otherwise induced by glutamate (35 mM) (Figure 1b). The fluorescent dye 10-*N*-nonyl-acridine orange (NAO) was applied to measure ROS generation at mitochondrial levels. This ROS generation is typically induced by glutamate^52,53^ and normally under the control of the TSPO.^3,5^ MGV-1 and MGV-2 (25 *μ*M) prevent cardiolipin oxidation indicative of ROS generation at mitochondrial levels otherwise caused by exposure of U118MG cells to glutamate (Figure 1c). The prevention of collapse of the ΔΨm and generation of ROS show that initiation of the mitochondrial apoptosis cascade is inhibited.

For several reasons, the concentration of 35 mM of glutamate was chosen: (1) as its effect should be lethal, it should be somewhat higher than can be observed in normal healthy tissue; (2) not deviate from what can be found in diseased brain tissue; (3) the level of cell death induced in our experiments should be amenable to up and down regulation by our drug treatment (for more details, see Discussion and Materials and methods section).

**Figure 2:**
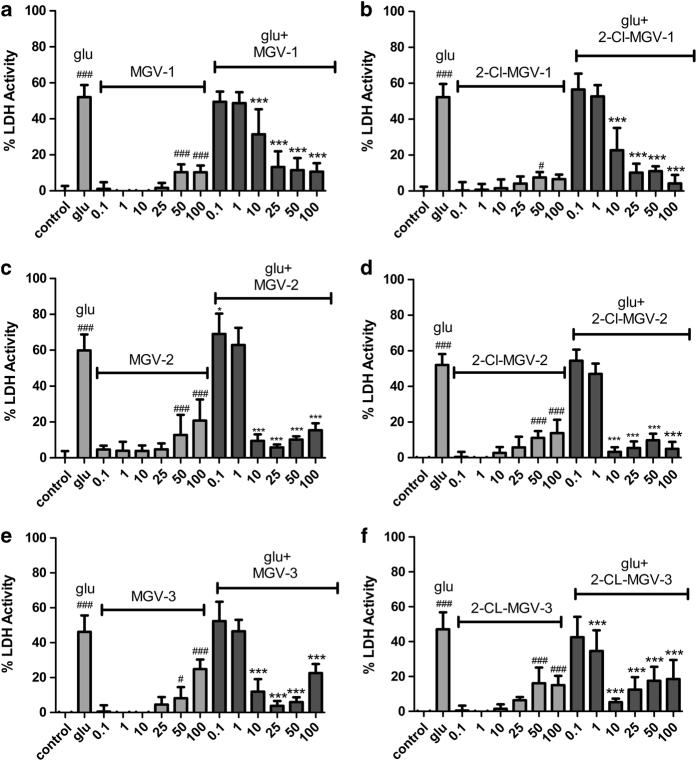
Protective effects of our quinazoline derivatives: MGV-1 (**a**); 2-Cl-MGV-1 (**b**); MGV-2 (**c**); 2-Cl-MGV-2 (**d**); MGV-3 (**e**); and 2-Cl-MGV-3 (**f**). In general, these compounds protect against glutamate-induced cell death of U118MG cells as measured by LDH activity. MGV-1, MGV-2, and MGV-3 protect against lethal effects of glutamate (**a, c** and **e**). MGV-2 and MGV-3 are already quite effective at 10 *μ*M (**c** and **e**), however, MGV-2 and MGV-3 also show undesired lethal effects at high concentrations (in particular 100 *μ*M), by themselves as well as in combination with glutamate (**c** and **e**). It appears that such undesired lethal effects can be reduced by adding a Cl substituent to the third carbocycle (**b, d** and **f**). In particular, 2-Cl-MGV-1 shows the least lethal effects when applied just by itself (**b**). Although 2-Cl-MGV-2 is very effective in protecting against glutamate-induced cell death at the concentrations 10, 25, 50, and 100 *μ*M, it still has undesired lethal effect at high concentrations when given just by itself (**d**). 2-Cl-MGV-3 has undesired lethal effects at 50 and 100 *μ*M both with and without glutamate exposure (**f**). In short, enhanced affinity for TSPO (see Figure 1) improves protective capability at 10 *μ*M, but enhances undesired lethal effects in particular at 100 *μ*M. Control, vehicle only (for glutamate and TSPO ligand); glu, glutamate. Further the x axis presents the treatments: control, glutamate, and application of compounds with their concentrations in *μ*M. The y axis presents the levels of cell death according to the formula for relative LDH release given in the Materials and methods. The results are expressed as mean values±S.D. One-way ANOVA followed by Bonferroni multiple comparison test was performed. *P<0.05, **P<0.01, ***P<0.001 treatment *versus* control. (*n*=12).

In Figure 2, applying lactate dehydrogenase (LDH), it is shown that at concentrations from 10 to 100 *μ*M, all the designed compounds in question can protect well to moderately well against the lethal challenge of 35 mM of glutamate to U118MG cells. Subtle differences can be seen in the protective effects in this group of six compounds (Figures 1 and 2). For example, *in vitro,* 2-Cl-MGV-2 protects excellently with all of these tested concentrations of 10, 25, 50, and 100 *μ*M, whereas the other compounds can be somewhat less effective at the relatively low concentration of 10 *μ*M or at the relatively high concentration of 100 *μ*M. Achieving a relatively high affinity of the compounds for TSPO, that is, MGV-3 and 2-Cl-MGV-3, does not improve the protective effects. 2-Cl-MGV-1 presenting a relatively low affinity for TSPO has the advantage of showing very little lethality by itself at any concentration (Figures 1 and 2).

**Figure 3:**
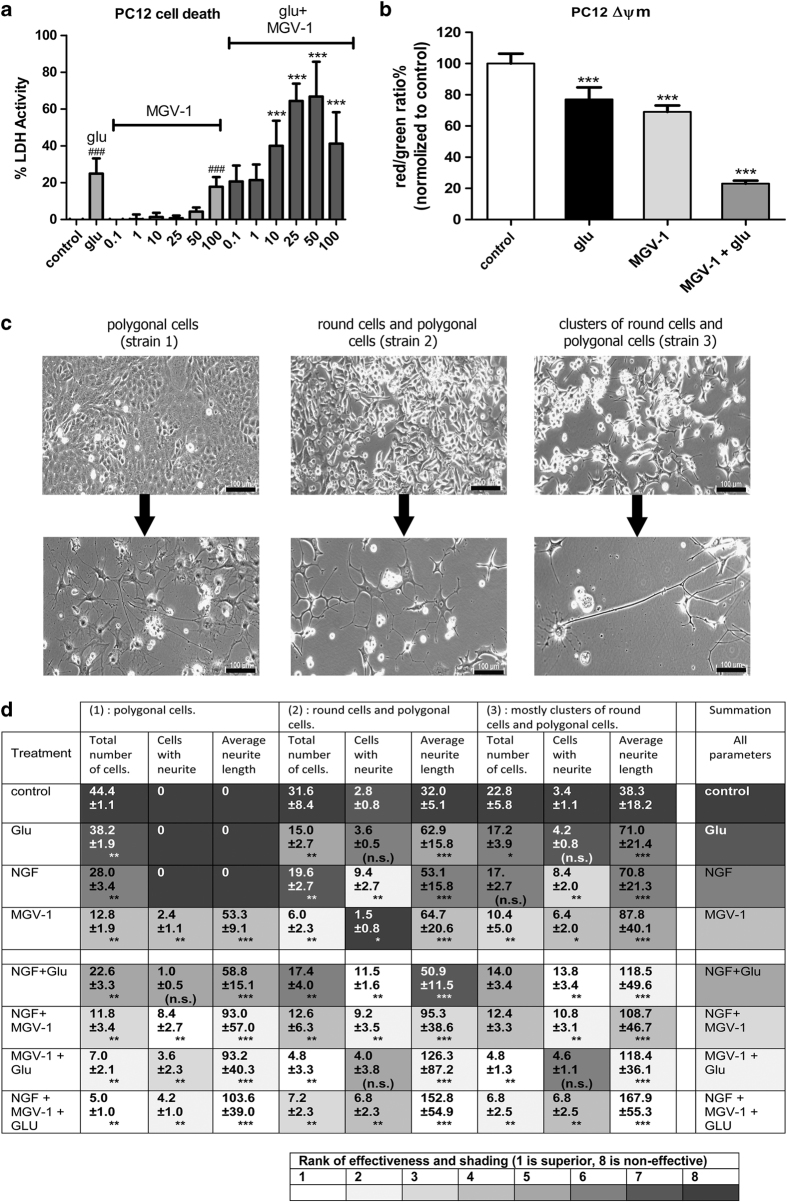
Effects of MGV-1 (and NGF and glutamate, as well as the various combinations of these three compounds) on PC12 cells regarding viability and differentiation. (**a**) As opposed to U118MG cells, together with 35 mM of glutamate, MGV-1 (10, 25, 50, 100 *μ*M as indicated at the x axis) induces enhanced cell death of PC12 cells, as compared with application of 35 mM of glutamate by itself. At 100 *μ*M, MGV-1 by itself can induce cell death of PC12 cells. (**b**) Separately and synergistically, MGV-1 (50 *μ*M) and glutamate (35 mM) induce collapse of the ΔΨm in PC12 cells. (**c**) Representative examples of different PC12 cell strains (see Materials and methods section): strain #1 (only flat, attached polygonal cells), strain #2 (round cells and polygonal cells), and strain #3 (floating clusters of round cells and a restricted number polygonal cells). MGV-1 can induce differentiation of various strains of PC12 cells. The top row of this **c** presents undifferentiated cells, and the bottom row of the **c** presents typical examples of differentiated cells of the three strains. As the morphologies of the undifferentiated cells of the three strains are different, the morphologies of these cell strains differentiated by our applications are also distinct. Neurite sprouting from strain #1 makes the cells appear star shaped (here differentiated by MGV-1+glutamate). Strain #2 gives rise to extended thin neurites (here differentiated by MGV-1+glutamate). Strain (3) gives rise to very long thin neurites (here differentiated by MGV-1+NGF). (**d**) Table: MGV-1, NGF, and glutamate, separately and combined, can induce neurodifferentiation of strains of PC12 cells presenting both spherical and polygonal cells (strains 2 and 3). The lower the total number of cells, the more the cells are differentiated (differentiated cells do not proliferate). The higher the number of cells with neurite (the hallmark of differentiation), the more the cells are differentiated. The longer the average neurite length, the more the cells are differentiated. The strain presenting only polygonal cells (strain #1), can be differentiated by MGV-1 by itself, whereas NGF and glutamate by themselves do not have this effect on cells of strain #1 (as demonstrated as 0 cells presenting a neurite, that is, neurites of 0 length). The shading in the Table for all treatments is according to rank each time in one column (see ‘key’ giving the shading for each of the 8 ranks). The murkiest shading for each parameter (total number of cells, cells with neurite, average neurite length) presents the lowest indication of differentiation (typically the control), the brightest shading indicates the most effective differentiation. Summing up the ranks of each row (presented in the most right-hand column) it was found, looking at the individual treatments of Glu, NGF, and MGV-1, that: MGV-1 works better than NGF works better than glutamate. Interestingly, measuring the percentages of differentiated cells as part of the total cell population remaining per plate, gives the exact same rank order of effectiveness. Regarding combinations of molecules: (MGV-1+Glu+NGF) works better than (MGV-1+Glu) works better than (MGV-1+NGF) works better than (NGF+Glu). Looking at each cell type regarding capacity of differentiation (comparing each parameter for each cell strain and each treatment), the rank order of capacity to differentiate is: Strain #3>Strain #2>Strain #1. Statistical significance following one-way ANOVA and *post hoc* Mann–Whitney: *P<0.05; **P<0.01; ***P<0.001. The scale bars in **c** are 100 *μ*M.

**Figure 4:**
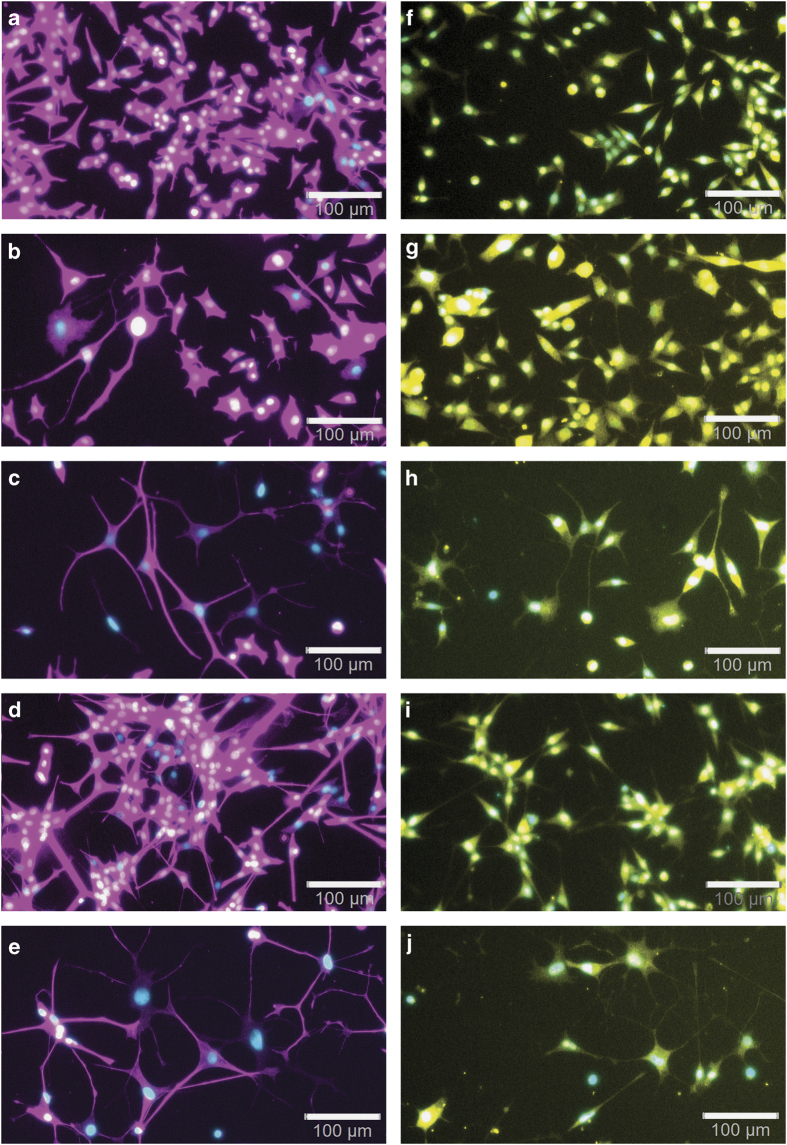
Localization of tubulin 3*β* (**a**–**e**) and NeuN (**f**–**j**) in cell bodies and neurites of differentiated PC12 (strain #3). This figure shows that our different protocols not only result in extensive sprouting and outgrowth of neurites of PC12 cells in culture (as shown in Figure 3), but also labeling of these cells with the neuronal markers tubulin 3*β* (magenta in **a**–**e**) and NeuN (yellow in **f**–**j**). The cell nuclei are labeled with DAPI (cyan in **a**–**j**). **(a**) Tubulin 3*β* labeling can be detected first of all in the cell bodies of the undifferentiated vehicle control PC12 cells (control). Inducing differentiation with MGV-1 (**b**), MGV-1 plus glutamate (**c**), NGF (**d**), as well as MGV-1 plus NGF plus glutamate (**e**) enhanced tubulin 3*β* labeling not only of the cell body but also intensely of neurites. (**f**) NeuN expression is indicated with yellow fluorescent immunocytochemical labeling of the cell bodies, both in the nuclei and the cytoplasm of undifferentiated cells (control). Nuclei and cytoplasm both are typical locations for NeuN.^91^ NeuN labeling can also appear in the neurites of cells differentiated with MGV-1 (**g**), MGV-1 plus glutamate (**h**), NGF (**i**), as well as MGV-1 plus NGF plus glutamate (**j**). NeuN labeling can also appear in the neurites. In undifferentiated as well as differentiated cells doubly labeled for DAPI and NeuN, the cell nuclei can appear whitish, indicating the presence of NeuN in the cell nuclei. The same is true Tubulin for cells doubly labeled for DAPI and tubulin. The scale bars in are 100 *μ*M.

Preliminary studies showed that these compounds can induce neurodifferentiation of PC12 cells. In particular, MGV-1 (50 *μ*M) was very effective in inducing extensive neuronal differentiation of PC12 cells, that is, outgrowth of neurites longer than the cell bodies' diameters. We first applied MGV-1 to a morphologically homogenous cell strain of PC12 cells, presenting a morphology of flat, polygonal, adherent cells (strain #1). A similar PC12 strain (ATTC CRL-1721.1) is also described by American Type Culture Collection (ATCC) (Manassas, VA, USA). These cell types are derived from the original PC12 source (ATCC CRL-1721), which presents clusters of floating spherical cells, but only a restricted number of flat, polygonal, adherent cells (polygonal cells). MGV-1 induced neurite outgrowth in all these three strains. Interestingly, although MGV-1 (50 *μ*M) prevented cell death induction by glutamate exposure (35 mM) of U118MG cells, this same treatment of PC12 cells with MGV-1 enhanced cell death induction by glutamate by threefold (Figure 3a). This was associated with a threefold enhancement of the incidence of collapse of the ΔΨm (Figure 3b). Interestingly, in the days following this treatment, the surviving PC12 cells of this strain #1 started to differentiate, that is, grew ever longer neurites, generally considered the hallmark of neurodifferentiation^23–25^ (Figures 3c and 4). Adding NGF and/or glutamate to the MGV-1 treatment enhanced the differentiation induced by MGV-1 (Figure 3d). However, in contrast to MGV-1, neither NGF nor glutamate applied by themselves or combined did result in appreciable neurodifferentiation of strain #1 (Figure 3d). To our knowledge, no publications report differentiation of the CRL-1721.1 PC12 cells with NGF (ATCC), although NGF is a standard application to differentiate CRL-1721 PC12 cells^23–25^ (Figure 3d).

We also applied our treatments to cells with the more standard looking PC12 cells, strains #2 and #3. We could differentiate these cells by NGF by itself as described previously by others (Figures 3d and 4). A new finding is that we could also differentiate these cells with glutamate by itself (Figures 3d and 4). Moreover, these cells could be differentiated by MGV-1 by itself as well. We also found that NGF, MGV-1, and glutamate can enhance each other's differentiating capabilities in these cells (Figure 3d). In applications with glutamate shown here, glycine is routinely applied. Noteworthy, however, omission of glycine does not modify the response of any of the cell strains (data not shown). Similarly, comparing the vehicle controls with and without alcohol (1% final concentration) also does not show a difference whatsoever (data not shown).

Our results further show that with virtually all parameters (Table in Figure 3d) strain #3 (compared with strains #1 and #2) shows the highest level of differentiation, that is, a larger percentage of the cells remaining in the culture dish is differentiated, and the average length of the neurites is the longest. As this cell line has the biggest proportion of spherical cells (Figure 3c), we assume that the spherical cell provide for the most extensive neurodifferentiation. MGV-1 by itself is more effective than NGF and glutamate by themselves regarding neurodifferentiation (Figure 3d). Regarding combinations of treatments to induce neurodifferentiation the rank order of effectiveness is: (MGV-1+NGF+glutamate)>(MGV-1+glutamate)**>**(MGV-1+NGF)>(NGF+glutamate) (Table in Figure 3d). Furthermore, each treatment that includes MGV-1 and/or NGF and glutamate is more effective than such treatments that do not include MGV-1.

**Figure 5:**
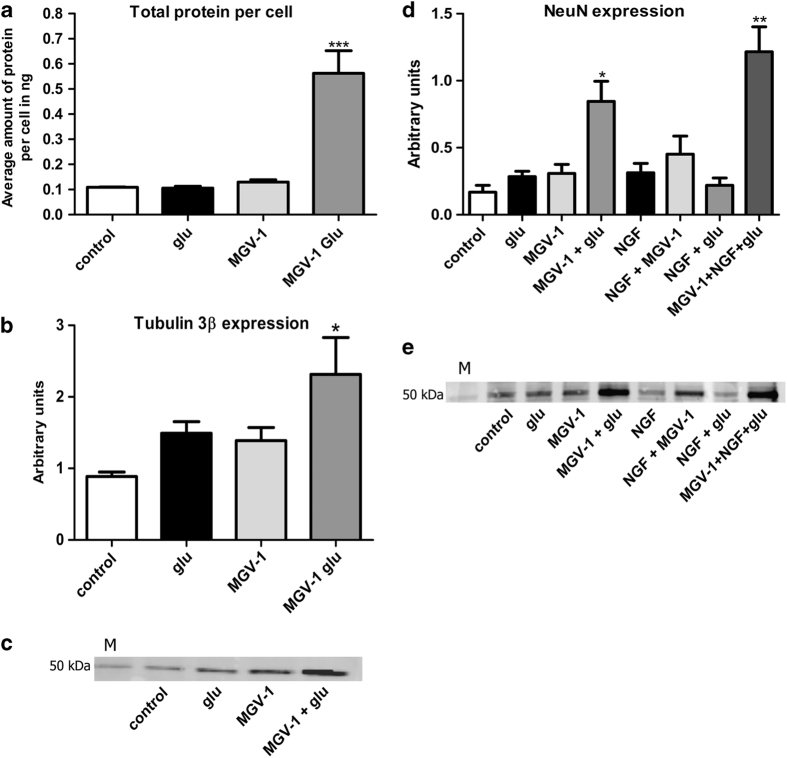
Neuroimmunochemical signs of differentiation of PC12 cells by our different exposure protocols, applied to Strain #1 (**a**–**c**) and Strain #3 (**d** and **e**) in comparison with their vehicle controls. (**a**) A bar graph showing protein content indicative of cell size of strain #1 differentiated by glutamate, MGV-1, and MGV-1+glutamate. MGV-1+glutamate enhances protein levels in these cells sixfold. (**b**) A bar graph of relative tubulin 3*β* expression in strain #1 cells differentiated by three different treatments (glutamate, MGV-1, and MGV-1+glutamate), compared with the vehicle control (undifferentiated cells). MGV-1+glutamate significantly enhances tubulin 3*β* expression in these cells. (**c**) Representative western blot assay of the effects on the expression levels of tubulin 3*β* of figures (**b**). (**d**) A bar graph showing significantly enhanced NeuN expression in cells of strain #3 differentiated by MGV-1+glutamate and by MGV-1+NGF+glutamate, compared with the vehicle control (undifferentiated cells). The other treatments shown (glutamate, MGV-1, NGF, NGF+MGV-1, NGF+glutamate) do not enhance NeuN expression significantly. (**e**) A representative western blot assay of NeuN expression in cells of strain #3 differentiated by our various protocols of Figure 4d. In (**b**) and (**d**), protein expression is given in arbitrary units (× 10^7^) as provided the ImageQuant LAS 4010 densitometer. Data presented as means±S.E.M. For 5**a** and 5**b** *n*=4, for 5**d** *n*=6. In all cases, statistical analysis by the Friedman ANOVA test, and Dunn's multiple comparison test as the *post hoc*. *P<0.05, **P<0.01, ***P<0.001 as compared with vehicle control (control). Control, vehicle only; glu, glutamate; M, molecular weight (50 kDa MW) markers for the western blots.

To further characterize neuronal differentiation, we separately assayed tubulin-3*β* (Figures 4a and e) and NeuN (Figures 4f and j) expression.^54,55^ We used nuclear labeling with DAPI as a counterstain to assay whether all cells would show tubulin, respectively, NeuN labeling. Immunofluorescence microscopy showed that our techniques provide intense tubulin-3*β* expression of cells of strain #3, both in cell bodies as well as neurites (Figures 4a and e). NeuN labeling was detected both in the nucleus and cytoplasm of cells of strain #3 (Figures 4f and j). The counterstain with DAPI showed that virtually all cells, under all conditions, show tubulin as well as NeuN labeling. The cells of strain #1 differentiated with MGV-1+glutamate typically were bigger than the non-differentiated control cells (Figure 3c), and contained six times more protein (Figure 5a). On top of this, western blots showed that tubulin expression was increased another threefold (Figures 5b and c). TSPO and *β*-actin expression, however, were not changed in differentiated polygonal cells relative to total general protein levels (data not shown). Also NeuN and GFAP did not show changes in the polygonal type (data not shown). Interestingly, strain #3 with predominantly spherical cells showed several fold increases in NeuN expression with western blot after MGV-1+glutamate (fivefold) and MGV-1+NGF+glutamate exposure (sevenfold) (Figures 5d and e), suggesting that the combination of MGV-1+glutamate is very conducive for NeuN expression in differentiated cells of strain #3.

### Animal studies

**Figure 6:**
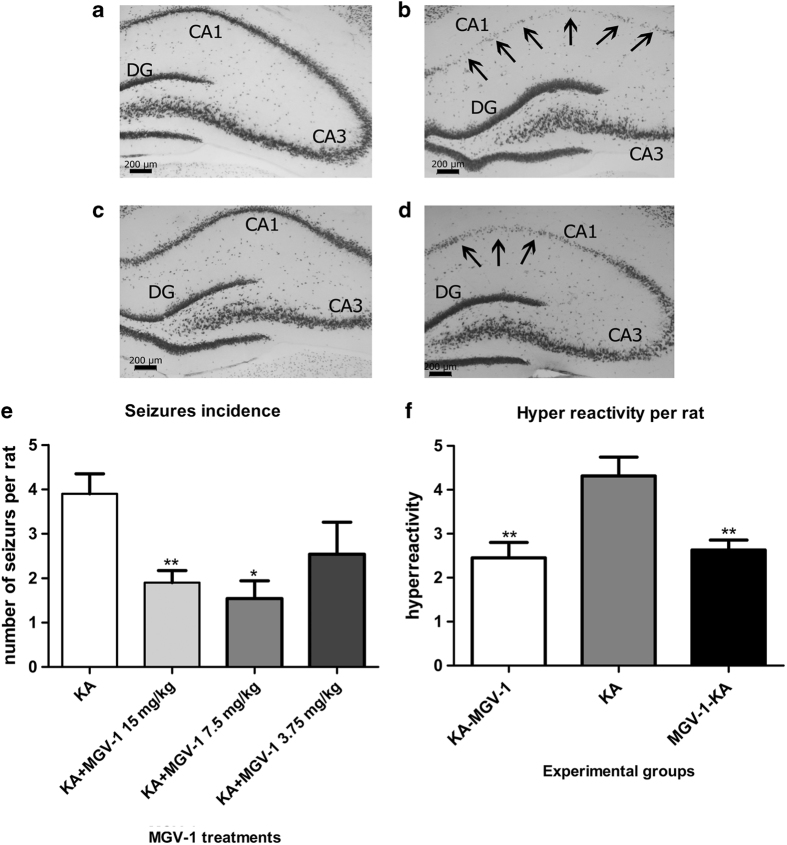
Neuroprotective effects by MGV-1 treatment of kainic acid-injected rats. Histology: representative effects of kainic acid injection and MGV-1 treatment on neuronal cells in rat hippocampus. (**a**) NeuN labeling in hippocampus of a vehicle control rat. (**b**) NeuN labeling in the hippocampus of a KA+vehicle-injected rats. The arrows indicate extensive neuronal death in CA1. (**c**) Neun labeling following MGV-1 pretreatment before KA injections. The hippocampus is virtually indistinguishable from vehicle control rat. (**d**) Neun labeling following MGV-1 post-treatment after KA injections. The damage indicated with arrows in CA1 is considerable reduced compared with KA+vehicle-injected rat. In summary, kainic acid causes major damage in CA1 of the hippocampus, which can be virtually prevented by pretreatment with MGV-1, and also attenuated by daily treatments with MGV-1 after kainic acid injection. CA3, cornu ammonis area 3; DG, dentate gyrus. Behavior: (**e**) Injections of 15 and 7.5 mg/kg of MGV-1, 2 h before kainic acid injections (pretreated), significantly prevent induction of seizures (reduction of seizure incidence by half). *P<0.05 and **P<0.01 *versus* KA. (**f**) Furthermore, MGV-1 treatment, 2 h before kainic acid injections (MGV-1-KA=pretreated), attenuates the incidence of the hyper reactivity in response to handling in the week after the kainic acid injections. Hyper reactivity typically is pronounced after kainic acid injections, in otherwise untreated animals (KA), likely due to the progressive effect of brain edema as a typical consequence of kainic acid injections that induce seizures.^42,43,46,48^ MGV-1 treatment starting 2 h after kainic acid injections that induce seizures (KA-MGV-1=post-treatment), and subsequently given every day in the week afterward, also reduces the incidence of the hyper reactivity in response to handling in the week after the kainic acid injections. Applying ANOVA and Wilcoxon matched-pairs signed rank test regarding the number of animals presenting hyper reactivity indicates a significant difference between MGV-1-treated mice and the vehicle-treated control. **P<0.01 both for pretreatment and posttreatment compared with vehicle control. *n*=9 for the experimental groups of **f**.

Applying double-blind studies showed that the tricyclic, quinazoline-based compounds had beneficial effects in animal models for various forms of brain damage, because of injury and disease. We applied MGV-1 to the rat model of systemic kainic acid injections that typically cause death of neurons and astroglia cells, associated with infiltration of microglia, in specific brain areas: hippocampus, amygdala, pyriform cortex, as well as subsequent edema throughout the brain.^43,45–48^ First of all, in comparison with vehicle control (Figure 6a), the damage typical for the hippocampus in area cornu ammonis area 1 (CA1) because of kainic acid (Figure 6b), was well attenuated by MGV-1 pretreatment (Figure 6c). Weeklong MGV-1 post-treatment also attenuated damage to area CA1, but less so than MGV-1 pretreatment (Figure 6d). The beneficial effects include prevention of cell death of neurons as determined with NeuN labeling (Figure 6). Microglia activation and cell death of astroglia was also observed (unpublished results). Comparable effects were observed in the amygdala and piriform cortex (unpublished results). The attenuation of brain damage by MGV-1 was also associated with a reduction in the emergence of seizures when MGV-1 was injected 2 h before the systemic kainic acid injections (Figure 6e). Injected 2 h after kainic injection, and subsequently once every day for 6 consecutive days, MGV-1 restores normal response to handling, that is, hyper reactivity in response to handling is returned to the level of normal responses that are typically observed in wild-type rats (Figure 6f). This may be indicative of reduction of edema.

**Figure 7:**
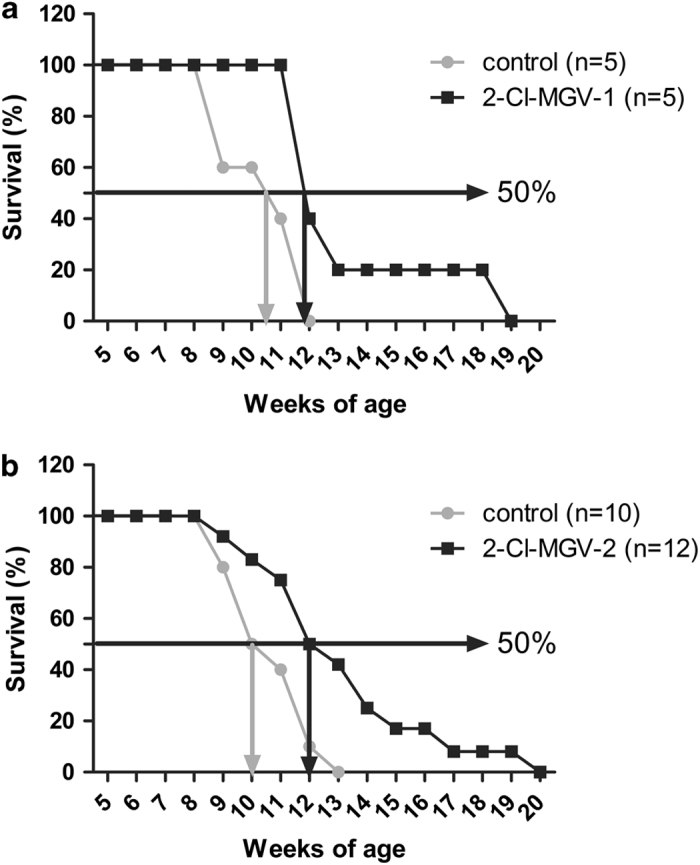
The MGV-1 derivatives 2-Cl-MGV-1 and 2-Cl-MGV-2 increase the lifespan of R6-2 mice, which are a transgenic animal model for Huntington disease. (**a**) The quinazoline-based, tricyclic compound 2-Cl-MGV-1 (which includes the Cl halogen substituent on the third rotatable carbocycle, that is, a halogenated MGV-1) increases median lifespan of R6-2 mice (*n*=5), compared with the vehicle DMSO-treated R6-2 mice (*n*=5). In more detail, 50% of untreated mice died before their 11th week, whereas 50% of the 2-Cl-MGV-1-treated R6-2 mice were still alive until the 12th week. The y axis presents the percentage of surviving animals per week. The x axis presents the number of weeks from birth. The 50% survival cut off is marked with a horizontal arrow. The week where the 50% survival cut off is reached is marked with a vertical arrow (gray for control; black for 2-Cl-MGV-1-treated mice). Applying Wilcoxon matched-pairs signed rank test regarding the number of surviving animals indicates a significant difference between the 2-Cl-MGV-1-treated R6-2 mice and the DMSO (vehicle)-injected R6-2 mice: *P*<0.01. Applying Mann–Whitney to each week of treatment shows that at the week of 40% survival of the control mice (week 12 of age), the difference between the 2-Cl-MGV-1-treated R6-2 mice and the vehicle-injected R6-2 mice is significant: *P*<0.05 for week 12. Linear regression applied to weeks 8–13 shows that the intercepts are not equal (F=11.7, *P*<0.01), that is, 2-Cl-MGV-1-treated R6-2 mice start dying significantly later than vehicle-injected R6-2 mice. The slopes are very equal (F=3.23, *NS*), that is, at any given point in time fewer treated animals have died than untreated. (**b**) The quinazoline-based, tricyclic compound 2-Cl-MGV-2 (which in addition to the halogenation of 2-Cl-MGV-1, includes asymmetrical side chains, methyl and ethyl) increases median lifespan of the R6-2 mice (*n*=12), compared with the vehicle-treated R6-2 mice (*n*=10, control). In more detail, 50% of vehicle-treated R6-2 mice died before their 10th week, whereas 50% of the 2-Cl-MGV-2-treated R6-2 mice were still alive until the 12th week. The y axis presents the percentage of surviving animals per week. The x axis presents the number of weeks from birth. The 50% survival cut off is marked with a horizontal arrow. The week where the 50% survival cut off is reached is marked with a vertical arrow (gray for control; black for 2-Cl-MGV-2-treated mice). Applying ANOVA and Wilcoxon matched-pairs signed rank test regarding the number of surviving animals indicates a significant difference between the 2-Cl-MGV-2-treated R6-2 mice and the DMSO (vehicle)-injected R6-2 mice: *P*<0.01. Applying Mann–Whitney to each week of treatment shows that at the week of 50% survival of the 2-Cl-MGV-2-treated R6-2 mice (week 12 of age) and in the week after that, the differences between the 2-Cl-MGV-2-treated R6-2 mice and the vehicle-injected R6-2 mice were significant *P*<0.05 for each of these two weeks. To determine whether the death rate of the vehicle-injected R6-2 mice is steeper than the death rate of 2-Cl-MGV-2-treated R6-2 mice we applied linear regression. Looking over the whole survival periods of both groups (F=12.5), and also over the restricted period from the week of diagnosis (week 8, after which the first animals have died) till all of the vehicle-treated R6-2 mice have died (week 13) (F=20.8), in both instances a significant difference between slopes is seen, *P*<0.01. The intercepts are not significantly different, meaning that the animals of the vehicle-treated and -untreated animals start dying from the same age. Thus, it appears that 2-Cl-MGV-1 is the superior agent for treatment of this animal model for Huntington disease.

Surprisingly, MGV-1 did not appear to have an effect in the transgenic mouse model (R6-2 mice) for the hereditary human disease of Huntington, regarding behavior and lifespan.^20^ Also the classical TSPO ligand PK 11195 did not show such effects. However, derivatives from MGV-1 considerably and consistently extended average lifespan of these mice by 20%. In particular, in Figures 7a and b the effects of 2-Cl-MGV-1 and 2-Cl-MGV-2 on R6-2 mice are shown. MGV-2 had effects reminiscent of those of 2-Cl-MGV-1 and 2-Cl-MGV-2, but less consistent (data not shown). Interestingly, behavioral data suggested a reduction of uncontrollable tremor activity of R6-2 mice treated with 2-Cl-MGV-1 and 2-Cl-MGV-2, indicative of positive effects on motor control (data not shown).

## Discussion

**Figure 8:**
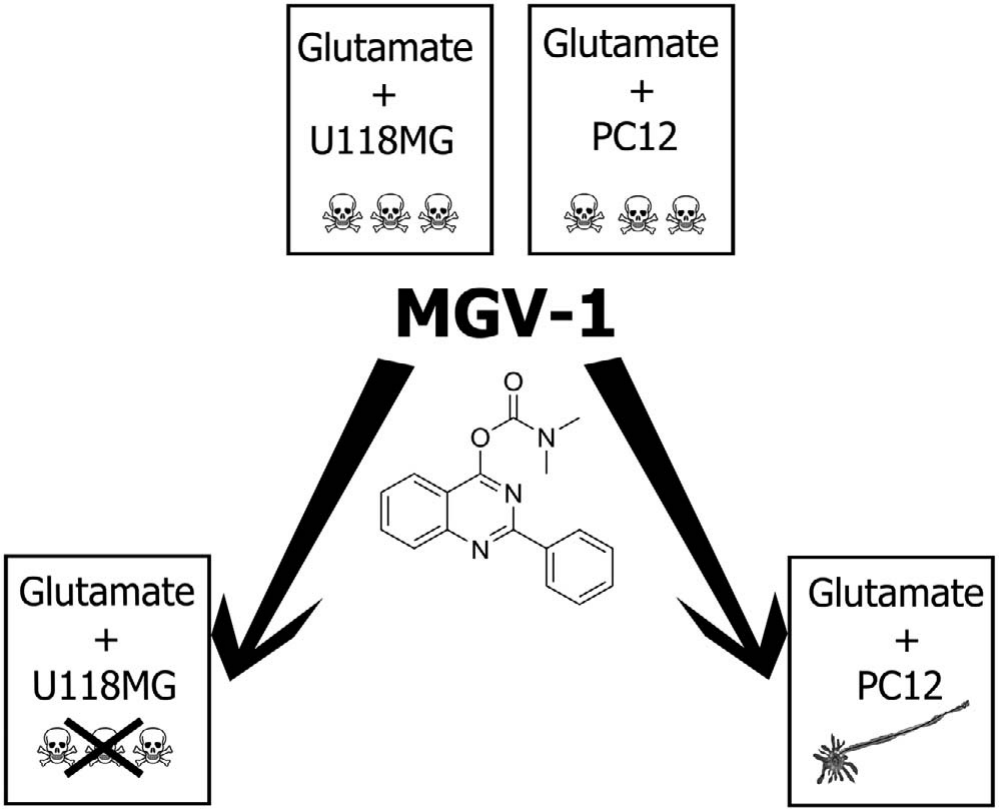
Summary of the effects of MGV-1 in cell cultures of U118MG and PC12 cells. Glutamate (35 mM) induces cell death of U118MG cells as well PC12 cells (skulls in top boxes). MGV-1 protects U118MG cells from glutamate-induced cell death (crossed out skulls in left-hand bottom box). In contrast, MGV-1 together with glutamate induces pronounced neuronal differentiation of PC12 cells (image of a mature neuron in most right-hand bottom box). As shown in Figure 3, MGV-1 also enhances cell death induction of glutamate of PC12 cells. Thus, MGV-1 appears to be able to regulate astrocytic integrity, neuronal differentiation, and weeding out of non-differentiating progenitor cells.^19,37^

Embryonic development of brain includes maintenance of a glial scaffold to support developing neurons.^56^ The neuronal development includes migration of enormous numbers of progenitor cells into specific target areas. Subsequently, millions of these cells reaching the target area die off, whereas only a very restricted number of progenitor cells (a few ten thousands) develop into mature neurons.^29^ In this study, the presented tricyclic compounds based on a quinazoline scaffold induced effects in cell culture that mimic those of embryonic brain development. This includes: (1) protecting cells of glial origin from cell death, (2) stimulating massive cell death of neuronal progenitor cells as induced by glutamate, and (3) stimulating extensive neuronal differentiation of the surviving neuronal progenitor cells (Figure 8). To emphasize, it appears that our relatively small tricyclic molecules basically can induce overall cell processes essential for brain development, as well as protect and cure adult brain damage. The latter was indeed demonstrated in our animal models.

Specifically, the molecules of this study are expanded from a bicyclic quinazoline scaffold and were designed to bind to the TSPO and to regulate TSPO-related functions. Thus, we assume that this is at least part of the way via which they regulate neurodifferentiation. It is known that TSPO regulates several functions, including but not restricted to: (1) programmed cell death, and (2) gene expression for various proteins essential for neurodifferentiation, such as growth factors and their receptors; enzymes for glutamate metabolism, and also glutamate receptors and glutamate transporters; proteins related to adhesion, migration, cell cycle, etc.^3–6,9,57^ This study showed that our compounds also can prevent initiation of events (collapse of the ΔΨm and generation of ROS) leading to the mitochondrial apoptosis cascade. It still can be assumed, however, that TSPO and its ligands, including our new compounds, can also regulate other cell death processes. A few studies have associated TSPO specifically with development/differentiation of progenitor cells to neuronal and glial cells.^13^

A classical agent to induce neuronal differentiation of PC12 cells is NGF.^23–25,58^ In this study, we report that apart from MGV-1 and NGF by themselves it is also possible to apply glutamate by itself for this purpose. As no difference is found with glycine added to the glutamate treatment, it seems that the *N*-methyl-D-aspartate (NMDA) receptor is not involved in differentiation induced by glutamate application. It is known that in healthy brain there is 5–15 mmol of glutamate per kg of tissue, depending on the region.^59–61^ Furthermore, in brain tissue around tumors, glutamate levels can be increased twofold.^62^ Intracellularly, it has been reported that glutamate concentration in synaptic vesicles can reach 60–250 mM.^63–65^ In synapses, glutamate levels typically are 1 mM or less.^66^ Keeping the physiological and pathological glutamate concentrations in cells and tissue in mind, and after generating dose-response curve from 0.1 to 100 mM of glutamate for U118MG cells, as well as PC12 cells (see Materials and methods section), we chose the concentration of 35 mM of glutamate for our study as its induction of cell death allowed for upregulation and downregulation. We then found that this concentration of 35 mM contributed substantially to neurodifferentiation typically induced by MGV-1, as well as NGF. We measured the maximal extent of neurodifferentiation (neurite length) 8 days after starting the treatments with our compounds. However, already within 3 days appreciable differentiation can be observed. Notably, our treatment protocols provide neurodifferentiation far more complete and extensive than generally presented in the research field of PC12 differentiation.^23–25,58^ Namely, with our most optimized methods, up to 100% of the population of cells is differentiated and average neurite length is 170 *μ*m. Maximal neurite length we have seen in this way was 670 *μ*m. The enhanced tubulin-3*β* and NeuN labeling suggests that the outgrowth of neurites indeed presents neuron-like features.^54,55^ For future studies, it would be interesting to test the effects of MGV-1 and related compounds on mouse progenitor cells,^13^ human progenitor cells,^67^ and primary neurons from developing brain.^68^ Also regarding PC12 cells, it appears to be worthwhile to apply MGV-1 and related compounds, as the differentiation procedure is extremely simple and productive.

As MGV-1 is able to differentiate the polygonal PC12 cells by itself (strain #1), whereas NGF and glutamate are not, it appears that in this model the MGV-1 (possibly via TSPO) is essential for differentiation in this PC12 strain #1. In this polygonal PC12 cell strain, NGF and glutamate can enhance the effect of MGV-1. The independent capabilities of NGF, MGV-1, and glutamate to differentiate spherical, as well as polygonal cells, and their synergistic interactions in these cells, suggest that these agents activate independent pathways that may converge to attain a final common effect regarding neurite sprouting in the original PC12 source strain. Further studies are needed to determine differences and commonalities in the pathways whereby MGV-1, glutamate, and NGF stimulate differentiation of PC12 cells, including the involvement of the TSPO. We have also found that the classical TSPO ligand PK 11195 by itself can differentiate PC12 cells (unpublished results).

Our animal studies indicate that our novel compounds are not only effective in cell culture but also are involved in neuroprotection and neuro-repair following brain damage because of neurotoxicity and a hereditary neurodegenerative disease. In short, the favorable effects of MGV-1 against the damaging effects of kainic acid reported here are: MGV-1 prevents the emergence of seizures, attenuates the hyper reactivity in response to handling that typically occurs in the days and weeks after the seizures, and attenuates occurrence of damage in the hippocampus, pyriform cortex, and amygdala. The MGV-1 treatments are effective when applied before kainic acid injections, indicating protective effects, and also when applied after kainic acid injections, implicating curative and restorative effects. Notably, the hyperactivity in response to handling is considered as a consequence of edema developing throughout the brain after excitotoxic hippocampal damage.^43,44,46,47^ Thus, MGV-1 appears to be able to counteract progressing brain edema, or at least its adverse effects. To summarize, MGV-1 appears to be able to counteract brain damage as well as the associated sensorimotor and behavioral deficits.^69^

To our surprise, MGV-1 did not work in an animal model for Huntington disease (R6-2 transgenic mice). However, derivatives of MGV-1 that in cell culture do protect better than MGV-1, can reduce locomotor aberrations and also increase the lifespan of R6-2 mice. It appeared that the addition of a Cl substituent to the third carbocycle of our compounds is very advantageous for the treatment of this transgenic mouse model. The death of the R6-2 mice is reminiscent of sudden unexplained death in epilepsy in humans that may include heart failure.^69^

To summarize, in cell culture, our new drugs regulate programmed cell death and induce neurodifferentiation (Figure 8). In the animal models for brain injury and disease, our new drugs: (1) prevent epileptic seizures, (2) improve locomotion, (3) normalize responsiveness, (4) maintain normal brain neurohistology, (5) reduce anxiety, (6) prevent heart failure associated with seizures, (7) extend lifespan, and, importantly, (8) present no adverse effects. In cell cultures, MGV-1 and related compounds prevent cell death of glial-like U118MG cells, whereas it weeds out undifferentiated PC12 cells that are not sent on a neurodifferentiation pathway.^69^ Thus, these tricyclic molecules by themselves can induce diverse processes that also occur during normal embryonic brain development and promote protective and restorative responses in adult brain tissue damage.

## Materials and Methods

### Research subjects

- U118MG cells were generously provided by Dr. G Bernhardt, University of Regensburg, Germany.^57^ These cells were originally obtained in 1994 from ATCC, and maintenance of morphology, karyotype, proliferation *in vitro* and *in vivo*, histology, and responses to the relevant cytostatics have been checked regularly since then until present.^6,70–72^
- Three different PC12 cell strains derived and modified from the original ATCC CRL-1721 PC12 cell source were generously provided by Dr. I Gozes (strain #1) (University of Tel Aviv, Israel), Dr. S Engelender (strain #2) (Technion – Israel Institute of Technology, Haifa, Israel), and Dr. I Perlman (strain #3) (Technion - Israel Institute of Technology, Haifa, Israel). The cells from strain #3 showed the typical morphology of PC12 cells, that is, clustered floating spherical cells accompanied by a restricted number of flat polygonal attached cells. The cells from strain #2 also showed clustered floating spherical cells accompanied by a relatively large number of flat polygonal attached cells. The cells from strain #1 only presented flat polygonal attached cells. This latter strain is similar in appearance to the ATCC CRL-1721.1 PC12 strain (own observations).
- Sprague–Dawley male rats 7 weeks of age were ordered from Harlan, Jerusalem, Israel (for a temporal epilepsy, neurodegeneration model induced by systemic kainic acid injection).
- R6-2 mice (~120 CAG repeats B6CBA-Tg(HDexon1)62 Gpb/3J) presenting a transgenic mouse model for Huntington disease, a hereditary neurodegenerative disease in humans, were obtained from the Jackson Laboratory (Bar Harbor, ME, USA) and bred. In particular, males with the genotype hemizygous for Tg(HDexon1)62 Gpb, and females from the wild-type background strain B6CBAF1/J, were bred according to the protocol of Jackson Laboratory.
- Tricyclic compounds based on a quinazoline scaffold were designed and prepared by us,^19,20^ for details see below.
- NGF (mouse NGF, 2.5S from Promega, Madison, WI, USA) was purchased via Biological Industries, Beit Haemek, Israel.
- Glutamate was purchased from Sigma, Rehovot, Israel.
- Kainic acid (KA) was purchased from Ascent Scientific (Bristol, UK).

### Materials

#### Chemical synthesis

For synthesis of the compounds designed for this study (MGV-1, 2-Cl-MGV-1, MGV-2, 2-Cl-MGV-2, MGV-3, and 2-Cl-MGV-3), all commercially available reagents were from Sigma-Aldrich (Rehovot, Israel) and used as received, unless otherwise stated.

#### Cell culture and viability assay materials

Materials for cell culture including media, serum, gentamycin, glutamine, penicillin, streptomycin, saline, Trypan blue, and trypsin were purchased from Biological Industries.

- Cytotoxicity detection kit (LDH) (Roche Pharmacuticals, Bazel, Switzerland).
- The fluorescent dye JC-1 was obtained from Calbiochem (Merck, Darmstadt, Germany).
- The NAO was obtained from Sigma.
- Cell culture vessels (Corning Life Sciences, Ramat-Gan, Israel).

#### Materials for binding assays and immunoblotting

Bradford solution for determination of protein concentration was obtained from Bio-Rad (Munich, Germany).

- Binding assay materials: [^3^H]PK 11195 (85.0 Ci/mmol) was purchased from New England Nuclear (Boston, MA, USA). 1-(2-Chlorophenyl-N-methyl-1-methylpropyl)-3-isoquinolinecarboxamide (PK 11195); was purchased from Sigma-Aldrich (Rehovot, Israel). CytoScintTM was obtained from MP Biomedicals (Costa Mesa, CA, USA); Whatman GF/C filters were obtained from Tamar (Mevaseret Zion, Israel).
- Protease inhibitor Complete was purchased from Roche Diagnostics (Mannheim, Germany). Ethylenediaminetetraacetic acid (EDTA) was purchased from JT Baker (Phillipsburg, NJ, USA).
- Ponceau staining solution was obtained from Sigma-Aldrich (Rehovot, Israel).
- Antibodies: anti-tubulin-3*β* antibody from mouse was obtained from Sigma-Aldrich (Rehovot, Israel); and anti-*β*-actin antibody (Santa Cruz Biotechnology, Santa Cruz, CA, USA) polyclonal anti-rat TSPO antiserum (from rabbit) was prepared in our laboratory, as previously described.^73^ Anti-human TSPO antiserum (from rabbit) was prepared in our laboratory;^57^ and also purchased from Abcam (from rabbit) (Zotal, Tel Aviv, Israel); and anti-neuronal nuclei (NeuN) (from rabbit) was purchased from Cell Signaling, Petach-Tikva, Israel; secondary antibodies (anti-rabbit and anti-mouse – IgG linked to horseradish peroxidase) were obtained from Amersham GE Healthcare (Buckinghamshire, England).
- Precision pre-stained standards (10–250 kDa) were from Bio-Rad Laboratories (Hercules, CA, USA).
- Keyhole limpet hemocyanin (maleimide-activated KLH) was obtained from Pierce (Rockford, IL, USA).
- Dried milk was obtained from Carnation (Glendale, CA, USA).
- Nylon-reinforced nitrocellulose membranes Nytran, S & S were obtained from TAMAR, Jerusalem, Israel.
- EZ-ECL was purchased from Bioconsult, Jerusalem, Israel.
- QIAGEN DNA EXTRACTION KIT, Eldan Electronic Instruments, Petach-Tikva, Israel.
- DNA polymerase kit (DreamTaq PCR Master Mix (2X), Thermo Scientific, Tamar Laboratory Supplies, Mevaseret Zion, Israel).

#### Standard chemicals

- Standard chemicals were purchased from Sigma-Aldrich, St. Louis, MO, USA, unless stated otherwise.

### Equipment

- The Spectrophotometer Zenyth 200 (ELISA reader) was from Anthos (Eugendorf, Austria).
- The Kinematica Polytron was from Brinkmann Instruments (Westbury, NY, USA).
- The flow cytometer used was the FACSCalibur, including its software, from Becton Dickinson (Mountain View, CA, USA).
- The Elisa reader Ceres UV 900 was from Bio-Tek (Burlington, VT, USA).
- The *β*-counter, a 1600 CA Tri-Carb liquid scintillation analyzer, was from Packard (Meriden, CT, USA).
- Surgery equipment was from Bar Naor (Ramat-Gan, Israel).
- The densitometric apparatus was ImageQuant LAS 4010 (GE Health Care Life Sciences, Rehovot, Israel).
- Thermal cycler (MJ Mini, Bio-Rad Laboratories, Rishon Le Zion, Israel).
- E-Gel PowerBase apparatus (Invitrogen, Life Technologies, RHENIUM, Modi'in, Israel)
- LAS-3000 luminescent image analyzer (Fujifilm, Tokyo, Japan).
- Hamilton–Kinder sensor in a soundproof ventilated apparatus (Kinder Scientific, Poway, CA, USA).
- Z1 inverted microscope (Zeiss, Oberkochen, Germany).

### Methods

#### Compound synthesis

The compounds have been described previously,^19,20^ and produced by us for this study according to state of the art methods. All reactions were performed under argon atmosphere in flame-dried glassware. Progress of the reactions was monitored by analytical thin layer chromatography (TLC) using glass plates precoated with silica gel with F254 indicator to measure absorbance at 254 nm wavelength. ^1^H and ^13^C NMR were recorded on 300 (75) MHz or 400 (100) MHz spectrometers. Chemical shifts are reported in p.p.m. (*δ*) units using tetramethylsilane as the internal standard. The following abbreviations were used to designate chemical shift multiplicities: s=singlet, d=doublet, t=triplet, q=quartet, m=multiplet. All first-order splitting patterns were assigned on the basis of the appearance of the multiplet. Splitting patterns that could not be easily interpreted are designated as multiplet (m). High resolution mass spectrum (HRMS) was obtained by Atmospheric Pressure Photoionization Source (APPI) method. Column chromatography was performed using Silica gel (230–400 mesh). 2-Arylquinazolin-4-ol (A in synthesis scheme below) was synthesized from *o*-aminobenzoate and corresponding aromatic aldehyde as described.^19,20,74^

#### General method for synthesis of 2-arylquinazolin-4-yl alkylcarbamate:


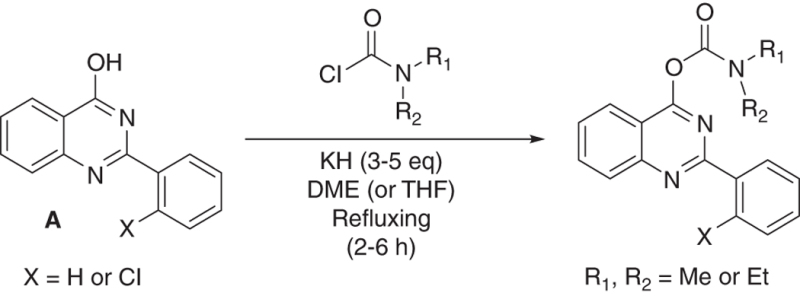


To a suspension of potassium hydride (6.75 mmol) under argon in 30 ml of dry 1,2-dimethoxyethane (DME) or dry tetrahydrofuran (THF) was added 2-arylquinazolin-4-ol (A in the synthesis scheme) (4.5 mmol) in one portion. The reaction mixture was stirred for 30 min at room temperature and subsequently, the carbamoyl chloride (7 mmol) was added to the reaction mixture. Reaction mixture was brought to reflux for 2–6 h till completion of reaction (TLC monitored). The reaction was quenched carefully with water (30 ml), followed by extraction with dichloromethane (3×50 ml). Combined organic phases were dried over MgSO_4_ and the solvents were evaporated under reduced pressure. The resulting crude product was purified by silica gel chromatography using hexane/ethylacetate as eluent (85/15), which was recrystallized with DCM/ethylacetate/*n*-pentane.

MGV-1:


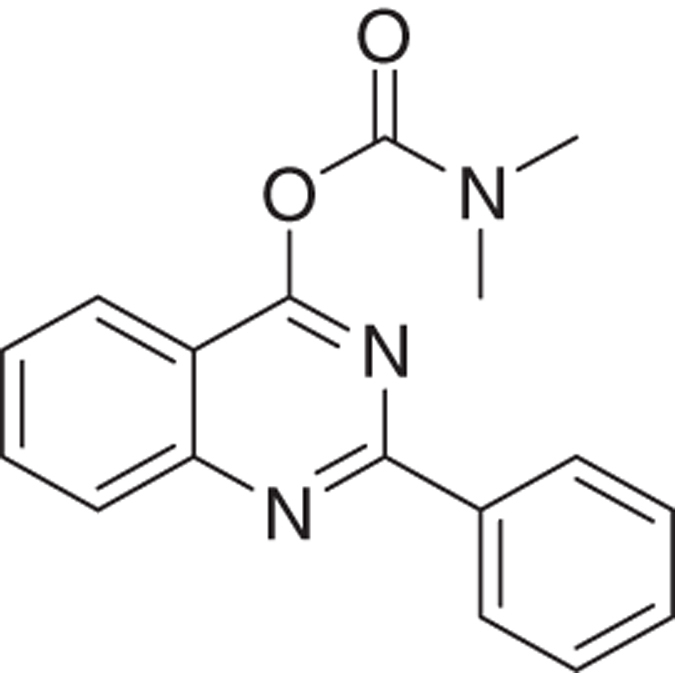


The title compound 2-phenylquinazolin-4-yl dimethylcarbamate (MGV-1) was obtained as colorless solid in 60% yield. ^**1**^**H NMR** (300 MHz, CDCl_3_) *δ* (p.p.m.): 3.15 (s, 3H), 3.17 (s, 3H), 7.49–7.44 (m, 3H), 7.56 (t, *J*=7.8 Hz, 1H), 7.86 (t, *J*=6.9 Hz, 1H), 8.07 (d, *J*=7.8 Hz, 2H), 8.53–8.50 (m, 2H). ^**13**^**C NMR** (75 MHz, CDCl_3_) *δ* (p.p.m.): 38.9, 117.9, 125.2, 129.0, 130.2, 130.3, 130.5, 132.5, 136.1, 155.1, 162.2, 165.9. **HRMS** (APPI): mass calcd for C_17_H_15_N_3_O_2_ [M+H]^+^: 294.1237; found: 294.1243.

MGV-2:


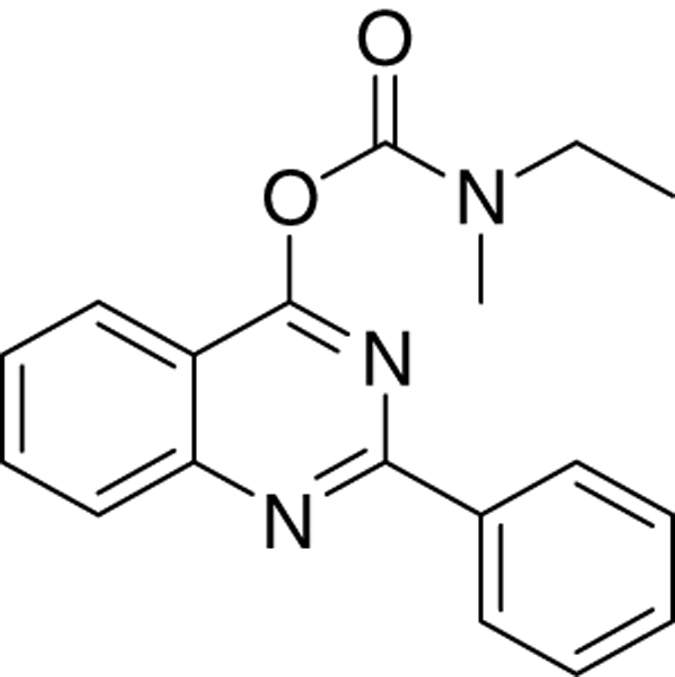


The title product 2-phenylquinazolin-4-yl ethyl(methyl)carbamate (MGV-2) was obtained as white solid in 65% yield. ^**1**^**H NMR** (300 MHz, CDCl_3_), rotamers observed in spectrum, *δ* (p.p.m.): 1.32 and 1.33 (2t, *J*=7 Hz, 3H), 3.12 and.3.13 (2 s, 3H), 3.50–3.59 (m, 2H), 7.45–7.50 (m, 3H), 7.54–7.59 (m, 1H), 7.85–7.90 (m, 1H), 8.04–8.09 (m, 2H), 8.51–8.55 (m, 2H).^**13**^**C NMR** (75 MHz, CDCl_3_), rotamers observed in spectrum, *δ* (p.p.m.): 12.23 and 13.40, 34.40 and 34.46, 44.55 and 44.84, 115.81 and 116.19, 123.25 and 123.29, 127.11 and 127.15, 128.28 and 12.32, 128.36, 128.56 and 128.59, 130.63, 134.21, 137.28, 153.12 and 153.20, 160.26 and 160.34, 164.05 and 164.14, 179.10. **HRMS** (APPI): mass calcd for C_18_H_18_N_3_O_2_ [M+H]^+^: 308.1394; found: 308.1397.

MGV-3:


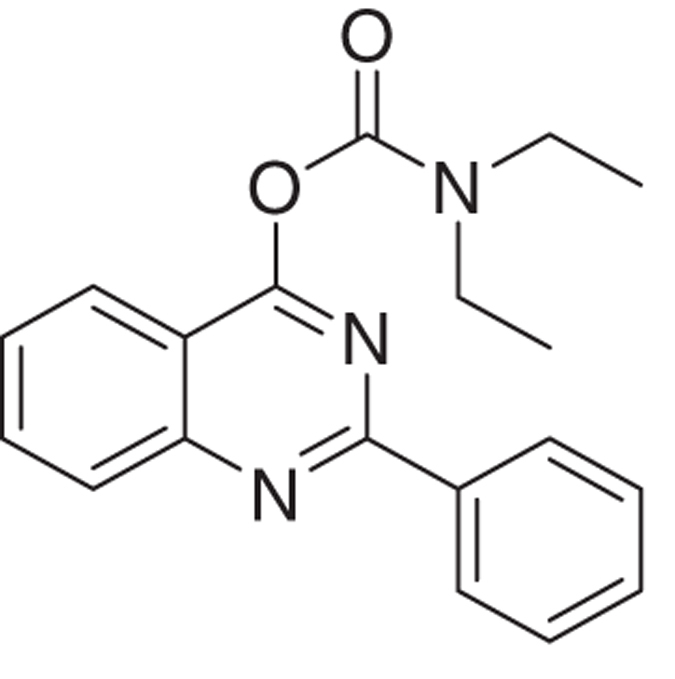


The title product 2-phenylquinazolin-4-yl diethylcarbamate (MGV-3) was obtained as white solid in 60% of yield. ^1^H NMR (400 MHz, CDCl_3_) *δ* 1.19–1.42 (m, 6H), 3.32–3.68 (m, 4H), 7.36–7.63 (m, 4H), 7.85 (t, *J*=6.7 Hz, 1H), 8.05 (d, *J*=8.3 Hz, 2H), 8.51 (dd, *J*=6.6 and 3.0 Hz, 2H). ^**13**^**C NMR** (100 MHz, CDCl_3_), rotamers observed in spectrum, *δ* (p.p.m.): 13.41 and 14.65, 42.90 and 43.00, 116.37, 123.61, 127.42, 128.61, 128.67, 128.91, 130.94, 134.49, 137.63, 151.82, 153.46, 160.63 and 164.57. **HRMS** (APPI): mass calcd for C_19_H_19_N_3_O_2_ [M+H]^+^: 322.1550; found: 322.1585.

2-Cl-MGV-1:


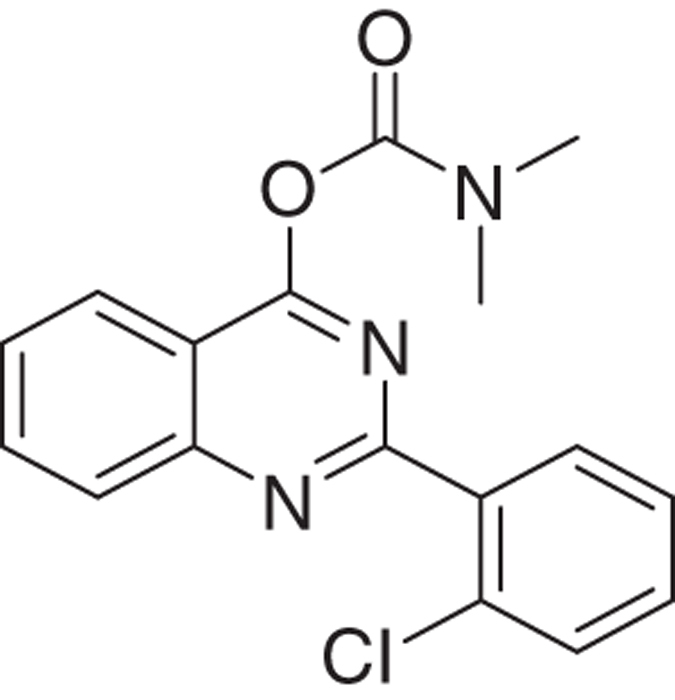


The title product 2-(2-chlorophenyl)quinazolin-4-yl dimethylcarbamate (2-Cl-MGV-1) was obtained as white solid in 47% of yield. ^**1**^**H NMR** (400 MHz, CDCl_3_) *δ* 3.10 (s, 3H), 3.16 (s, 3H), 7.31–7.42 (m, 2H), 7.44–7.51 (m, 2H), 7.65 (dd, *J*=8.6 and 7.2 Hz, 1H), 7.80–7.87 (m, 1H), 7.92 (t, *J*=7.8 Hz, 1H), 8.05–8.21 (m, 2H). ^**13**^**C NMR** (100 MHz, CDCl_3_) *δ* 37.34, 116.20, 123.61, 127.00, 130.60, 130.77, 132.26, 133.26, 134.78, 137.73, 152.11, 153.04, 161.17, 164.11. **HRMS** (APPI): mass calcd for C_17_H_14_ClN_3_O_2_ [M+H]^+^: 328.0847; found: 328.0864.

2-Cl-MGV-2:


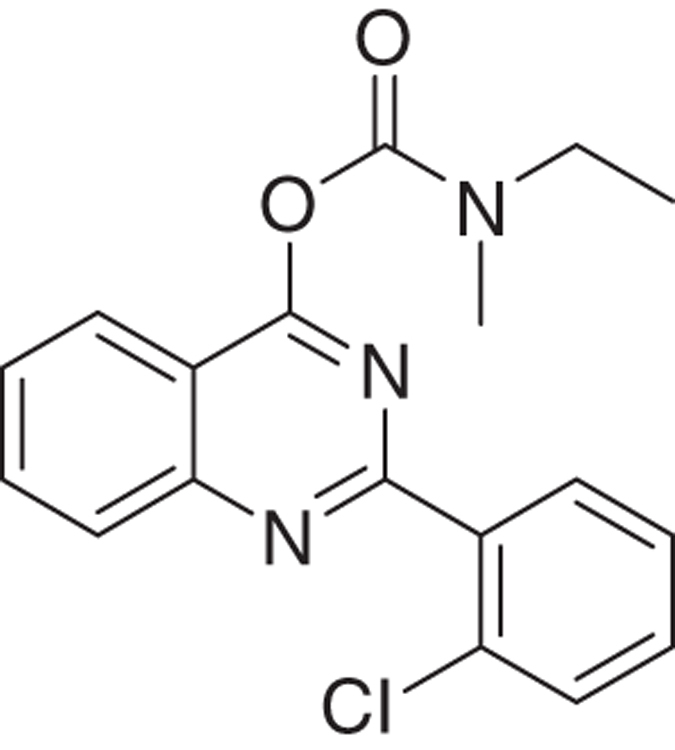


The title product 2-phenylquinazolin-4-yl ethyl(methyl)carbamate (MGV-2) was obtained as white solid in 65% of yield. ^**1**^**H NMR** (400 MHz, CDCl_3_), rotamers observed in spectrum, *δ* (p.p.m.): 1.17–1.32 (m, 3H), 3.04 and 3.08 (2 s, 3H), 3.38–3.58 (m, 2H), 7.33 (dd, *J*=7.0 and 3.2 Hz, 2H), 7.40–7.50 (m, 1H), 7.61 (dd, *J*=8.6 and 7.4 Hz, 1H), 7.78–7.84 (m, 1H), 7.88 (dd, *J*=9.4 and 7.2 Hz, 2H), 8.08 (dd, *J*=9.5 and 7.2 Hz, 2H). ^**13**^**C NMR** (100 MHz, CDCl_3_), rotamers observed in spectrum, *δ* (p.p.m.): 12.43 and 13.65, 34.64 and 34.74, 44.82 and 45.12, 116.07 and 116.36, 123.52 and 123.57, 126.92, 128.13 and 128.19, 128.60, 130.52, 130.71, 132.23, 133.23, 134.68, 137.70 and 137.72, 151.71 and 151.92, 152.95 and 152.99, 161.09 and 161.15, 164.11 and 164.19. **HRMS** (APPI): mass calcd for C_18_H_16_ClN_3_O_2_ [M+H]^+^: 342.1004; found: 342.1010.

2-Cl-MGV-3:


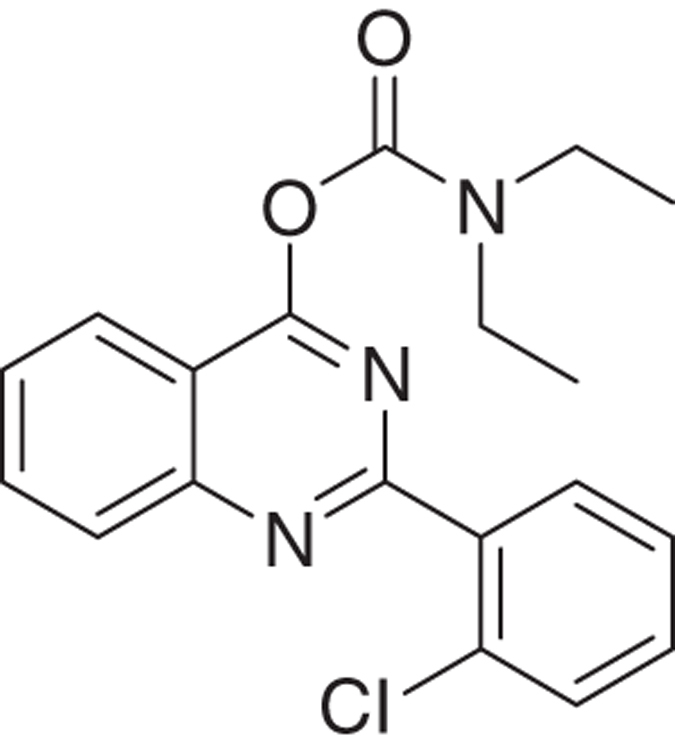


The title product 2-(2-chlorophenyl) quinazolin-4-yl diethylcarbamate (2-Cl-MGV-3) was obtained as white solid in 69% of yield. ^**1**^**H NMR** (400 MHz, CDCl_3_) *δ* (p.p.m.): 1.02–1.55 (m, 6H), 3.23–3.76 (m, 4H), 7.26–7.40 (m, 2H), 7.41–7.53 (m, 1H), 7.57–7.72 (m, 1H), 7.75–7.97 (m, 2H), 8.01–8.27 (m, 2H). ^**13**^**C NMR** (100 MHz, CDCl_3_), rotamers observed in spectrum, *δ* (p.p.m.): 13.25 and 14.55, 42.85, 116.27, 123.50, 126.86, 128.10, 128.55, 130.47, 130.68, 132.23, 133.21, 137.66, 151.55, 152.92, 161.08, 164.20. **HRMS** (APPI): mass calcd for C_19_H_18_ClN_3_O_2_ [M+H]^+^: 356.1160, found: 356.1167.

#### Protein level measurements

Protein levels of cell homogenates for binding studies and western blot assays were measured by the Bradford method^75^ using bovine serum albumin (BSA) as a standard. Also the amount of protein per cell was calculated.

#### TSPO binding for quality control of novel compounds:

Binding assays of [^3^H]PK 11195, including displacement studies were done as previously described.^76^ The reaction mixtures for the binding assays contained 400 *μ*l of homogenized rat kidney membranes (5 mg of homogenate/ml) and 25 *μ*l of [^3^H]PK 11195 (2 nM final concentration)^76^ in the absence (total binding) or presence of various concentrations (10^-10^ M–10^-5^ M) of the synthesized compounds described in this study. After incubation for 60 min at 4 °C, samples were filtered under vacuum over Whatman GF/C filters and washed three times with 5 ml of 50 mM Tris-HCl buffer, pH 7.4. The filters were incubated in CytoScint (MP Biomedicals) and radioactivity was measured after 12 h with a 1600 CA Tri-Carb liquid scintillation analyzer (Packard). Inhibitory constant (Ki) values were calculated by the equation Ki=IC50/(1+C/Kd), where C=[^3^H] PK 11195 concentration, IC50=concentration causing 50% inhibition of [^3^H]PK 11195 binding and Kd=2 nM (from Scatchard analysis of [^3^H]PK 11195 binding to kidney membranes).

### Maintenance and use of research subjects

#### Cell cultures

Cell cultures were done under sterile conditions at 37 °C under air with 5% CO_2_ using a humidified incubator:

##### U118MG cells

Cells of the human glioblastoma cell line, U118MG, were incubated in MEM EAGLE medium supplemented with 10% of heat-inactivated fetal bovine serum (FBS), 0.5 *μ*l/ml gentamicin, and 2% L-glutamine.^57^

##### Undifferentiated PC12 cells

PC12 cells (cell line from rat adrenal gland, Phaeochromocytoma) were cultured with complete DMEM medium containing 8% heat-inactivated FBS, 8% horse serum, 1 *μ*l/ml penicillin–streptomycin, and 1% L-glutamine.

#### Exposure to glutamate to induce cell death and its modulation by MGV-1

(1) For U118MG cells, glutamate at a concentration of 35 mM was applied with or without MGV-1. As discussed, glutamate levels in normal healthy brain tissue can average 15 mM, and these levels can be doubled in diseased brain tissue, whereas in cell organelles glutamate levels can be as high as 250 mM (as discussed). The concentration of 35 mM of glutamate *in vitro* provides a level of cell death that can be upregulated as well as downregulated by our application of TSPO ligands, as was determined *a priori* by dose-response analysis. In more detail, U118MG cells were seeded, 2×10^5^ cells per well of 6-well plates and 6×10^3^ cells per well of 96-well plates. Twenty-four hours after seeding, U118MG cells were pretreated with MGV-1 (or one of the other compounds) at various concentrations from 0.1 nM–100 *μ*M. Twenty-four hours after the pretreatment, the cells were exposed to 35 mM of glutamate. In all, 25 *μ*M as well as 50 *μ*M of MGV-1 were found to be most effective for protection against cell death (Figure 2), and were applied for subsequent experiments. The negative control was vehicle, that is, ethanol with a final concentration of 1%. The application of ethanol of this concentration was compared *a priori* with no ethanol application, and showed no effect for the various assays used. The positive control was exposure to 35 mM of glutamate alone (with 200 *μ*M of glycine).

(2) For PC12 cells (as for U118MG cells), dose-response assays of the lethal effects of glutamate on PC12 cells established 35 mM as an optimal concentration for glutamate for induction of LDH activity in the medium in the range of 20–40% as compared with the effect of the positive control, 1% Triton X-100, the latter which is defined as causing 100% of LDH release in the medium. As we did not want to work with marginal effects, but effects that can be up or downregulated by various treatments, we applied 35 mM of glutamate.

(3) Dose-response curves for MGV-1 as a modulator of cell death of PC12 cells were established using 0.1 nM–100 *μ*M of MGV-1 (50 *μ*M was found to be an optimal enhancer of cell death induced by glutamate). Importantly, this same treatment induced also differentiation of PC12 cells.

#### Differentiation of PC12 cells

For our newly developed differentiation method by MGV-1 in combination with glutamate, PC12 cells were plated onto poly-L-lysine coated culture plates and Petri dishes at a relatively low density (5.2×10^3^ cells/cm^2^ for six-well plates and 3.6×10^3^ cells/cm^2^ for Petri dishes), in DMEM medium containing 8% heat-inactivated FBS and 8% horse serum. Twenty-four hours after plating, MGV-1 was added (50 *μ*M of MGV-1 was found to be very effective). Twenty-four hours after this pretreatment, the medium was replaced with DMEM medium containing 0.5% heat-inactivated FBS and 0.5% horse serum (starvation medium) supplemented with MGV-1 together with glutamate (35 mM of glutamate was proved to be effective, see further below). Exposure to vehicle alone (culture medium with 1% of ethanol) was used as the control. (1% of ethanol by itself did not induce differentiation or cell death.) Six to 7 days after treatment, cells were collected for assays (see diagram below).


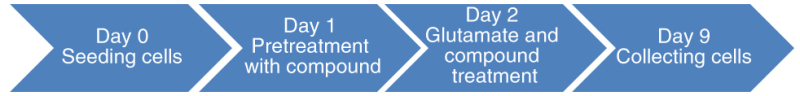


Basic diagram of temporal sequence of procedures applied to induce neurite sprouting of PC12 cells

During these 6–7 days, medium was not replaced. For differentiation by MGV-1 by itself, glutamate was omitted from the procedure. For differentiation by glutamate by itself, MGV-1 was omitted from the procedure. Glycine (200 *μ*M) was typically added to glutamate exposure, but could also be omitted as it had no effect for differentiation. With all treatments, cell cultures were subjected to daily microscopic evaluation and verification.

For differentiation of PC12 cells by NGF,^23^ applied for comparisons with differentiation by glutamate and MGV-1, PC12 cells were plated in DMEM medium containing 8% FBS and 8% horse serum. Twenty-four hours after plating, the medium was replaced with starvation medium containing 100 ng/ml of NGF. Twenty-four hours after adding NGF, the medium was replaced again with starvation medium containing 100 ng/ml of NGF. Six to 7 days after treatment, cells were collected for assays. During these 6–7 days, medium was not replaced. For additional assays, NGF treatment was combined with MGV-1 and/or glutamate. With all treatments, cell cultures were subjected to daily microscopic evaluation and verification.

#### Assays for cell culture studies

##### Viability assays

These assays were performed to learn the effects of our various treatments (see above) on cell viability and cell death of U118MG cells and PC12 cells.

##### Microscopic analysis

Before other assays, cell morphology, including rounding and blebbing, cell fragmentation, sprouting of neurites, etc. was analyzed with an inverted microscope to recognize apoptosis, necrosis, neurodifferentiation, etc.

##### Trypan blue exclusion assay

This traditional assay includes cell counts. Viable cells exclude Trypan blue, whereas nonviable cells absorb the dye because of impaired plasma membranes and appear blue.

##### LDH cytotoxicity assay

Wells of 96-well plates were with 6000 cells per well and treated according the protocol presented above. We used the Cytotoxicity Detection Kit (LDH) (Roche Pharmacuticals) according to the manufacturer’s instructions. When cell plasma membranes are damaged, the cytoplasmic enzyme LDH is released into the medium. The amount of formazan formed is proportional to the amount of LDH released.^77^ The resulting color intensity is proportional to the number of damaged cells. Absorbance at 492 nm with reference at 690 nm was measured with the Spectrophotometer Zenyth 200 (Anthos) and the results were calculated and normalized according to the formula presented by the manufacturer.

#### Mitochondrial assays

As mitochondria are important for virtually all life essential cell functions, including cell death as well as differentiation, and even regulation of gene expression, related events taking place at mitochondrial levels were assayed.

##### Metabolism assay with XTT

We used the Cell proliferation-XTT based assay kit (Biological Industries), as described previously.^72^ The 2,3-bis[2-methoxy-4-nitro-5-sulphophenyl]-2H-tetrazolium-5-carboxyanilide inner salt (XTT) assay is based on reduction of XTT by mitochondrial dehydrogenases of viable cells yielding an orange formazan product. Absorbance at 490 nm with reference at 680 nm was measured with the Spectrophotometer Zenyth 200 and results were normalized to control.

##### ROS assay with NAO

Cardiolipin is present in the inner mitochondrial membrane, at the contact sites with the outer mitochondrial membrane. By application of NAO, we assayed cardiolipin peroxidation as an indication of mitochondrial ROS generation, as described previously.^71^ NAO binds with high affinity to non-oxidized cardiolipin in a 2 : 1 ratio, but with less affinity to oxidized cardiolipin, the latter which is reflected by lower levels of green NAO fluorescence.^78^ For our study, cells were collected and centrifuged at 1200×*g* for 5 min, 4 °C. Cell pellets were re-suspended in 0.5 ml of 0.1 *μ*M NAO and incubated for 30 min at 37 °C in the dark. Then, the cells were washed once with 0.5 ml PBS and transferred into 5 ml FALCON FACS tubes and analyzed with the aid of a BD FACSCalibur flow cytometer using CellQuest software (BD Biosciences, Franklin Lakes, NJ, USA).

##### Mitochondrial transmembrane potential (Δψm) assay

JC-1 was used to assay changes in Δψm, as described previously.^71,79^ Owing to charges distributed along JC-1 molecules, they pass unimpaired (charged) mitochondrial membranes and enter the mitochondrial matrix. There the J-molecules form J-aggregates, which emit at 590 nm (orange–red fluorescence). When the Δψm collapses, JC-1 does not accumulate within the mitochondria, remaining in the cytoplasm in its monomeric form. The monomers emit at 527 nm (green fluorescence). A decrease in the red–green fluorescence intensity ratio is an indication for mitochondrial depolarization. As a positive control, the proton ionophore carbonyl cyanide m-chlorophenylhydrazone was used as described previously.^71^ Cells were collected and prepared for suspension in 10 *μ*g/ml of JC-1 solution in PBS. After incubation at 37 °C for 30 min in the dark, the cells were washed with 0.5 ml of PBS, and the cell suspensions were analyzed with the BD FACSCalibur flow cytometer.

#### Neuronal differentiation assays

##### Neurite length

The major and determining indication of neural differentiation of PC12 cells is neurite development.^23,80^ Equal sized digitized images of live PC12 cells exposed to MGV-1, glutamate, and NGF to induce neurite sprouting in our study were captured under phase contrast illumination with a Zeiss observer Z1 inverted microscope linked to a Hamamatsu Orca-R2 (Hamamatsu City, Japan) black and white camera. Images of five fields per plate were captured applying objective ×10. The number of differentiated cells was determined by visual examination of the captured fields. In particular, a line was drafted at half height of the image and the cell bodies crossed by the line were further analyzed regarding differentiation. The length of the line always was 841 *μ*m. Cells with at least one identifiable neurite with a length at least equal to the cell body diameter were scored, and their number expressed as a percentage of the total number of cells crossed by said line. In addition, for each of these cells, the length of the longest neurite was determined (using AxioVision Image software, Zeiss). Data collection from several timely separated replicates of PC12 differentiation was done and reproducibility established.

##### Western blot

The relative protein levels of TSPO and other proteins related to neurodifferentiation (including *β*-actin, NeuN, tubulin-3*β*), were assayed by immunoblotting with the aid electrophoresis through 12% SDS-polyacrylamide gel, according to previously described methods.^71^ *A priori*, general protein levels were measured by the method of Bradford using BSA as a standard.^75^ After electrophoresis, the gels were subjected to electrophoretic transfer to nitrocellulose membranes. Then, the membranes were blocked for 1 h in 5% non-fat milk in 62.5 mM sodium phosphate buffer, pH 7.4, containing 100 mM NaCl, and 0.1% (v/v) Tween-20 (PBS-T) and then incubated for 2 h or overnight at 4 °C with the appropriate antibodies: anti-tubulin-3*β* 1 : 10 000, anti-NeuN 1 : 1000, anti *β*-actin 1 : 15 000, and anti-TSPO 1 : 10 000.^73^ After washing with TBS-T, the membranes were incubated with IgG secondary antibody linked to horseradish peroxidase (anti-rabbit IgG 1 : 20 000, anti-mouse IgG 1 : 10 000, as appropriate, from Jackson Immunologicals, West Grove, PA, USA) in TBS-T at room temperature. Binding of antibodies to the membrane was detected with the EZ-ECL-detection reagent (Biological Industries), using the ImageQuant LAS 4010 apparatus and Totallab Quant quantitative image analysis of Totallab (Newcastle upon Tyne, England).

##### Immunocytochemical staining

Cells cultured for microscopic analysis of neurodifferentiation on six-well plates were fixed using paraformaldehyde 4% for 10 min, after 8 days of neurodifferentiation procedure. Then, 5% [w/v] BSA in PBS (blocking solution) was added for 1 h. Primary antibodies for our proteins of interest, potentially involved in neurodifferentiation (e.g., anti-tubulin-3*β* 1 : 10 000) and anti-NeuN 1 : 500), diluted in blocking solution were added at 4 °C for overnight incubation. The appropriate secondary antibodies (Cy3 AffiniPure Donkey Anti-Mouse and Donkey Anti-Rabbit IgG H&L (Alexa Fluor 488, preadsorbed) from Jackson ImmunoResearch, West Grove, PA, USA), were added for 1 h at room temperature. The plates were mounted using DAPI fluoromount-G (Southern Biotech, Birmingham, AL, USA), which also stains cell nuclei, and visualized using a Zeiss observer Z1 inverted microscope.

### Animals

All use and treatment of the rats and mice was approved by the local Institutional Review Committee of the Technion – Israel Institute of Technology (Haifa, Israel) following government guidelines. This included kainic acid injections of Sprague–Dawley rats, and the behavioral and histological assays regarding the treatments to attenuate the injurious effects of kainic acid and their consequences. It also included the breeding of R6-2 mice, their genotyping, the behavioral assays, the observations of their well-being and lifespan, and in particular the treatments to attenuate the negative effects of this hereditary disease. In particular, all behavioral observations were done double blind: first of all, the technician/observer did not know which treatments applied to which rats, and the experimenter was not informed of the results until the assay in question was concluded. We chose these animal models because various compounds binding to the TSPO (e.g., PK 11195, Ro5 4864) were shown previously to have protective effects in excitotoxic models for temporal lobe epilepsy and striatal damage as part of Huntington disease.^48,81,82^

#### Seizure induction in rats by kainic acid

Rats were obtained at an age of 7 weeks (Harlan). They were then kept individually in sterile cages at the animal facilities at the Rappaport Institute of the Medical Faculty of the Technion. The cages were kept in air-conditioned rooms, at a temperature of around 24 °C with a 12-h light–dark cycle. The rats were acclimatized for a week before the experiments.

Epileptic seizures in rats, including gnawing, wet dog shakes, head bobbing, forepaw abduction, rearing, forelimb clonus, loss of balance, and convulsions were induced by systemic injections of kainic acid and scored as previously described.^48,83–85^ Such induced seizures typically are accompanied by neurodegeneration in the hippocampus, amydala, pyriform cortex, followed by edema in the diencephalon, and mesencephalon.^43,44,86^ To test the effects of pretreatment with MGV-1 on the consequences of kainic acid injections, first of all MGV-1 was dissolved in vehicle (10% (v/v) dimethylsulfoxide (DMSO) in sesame oil). After that, 8 weeks old rats received an intraperitoneal injection of 15, 7.5, or 3.75 mg of MGV-1 (per kg/rat), according to previous methods.^48^ Kainic acid (9 mg/kg/rat), dissolved in phosphate-buffered saline obtained from Sigma-Aldrich (Rehovot, Israel), pH 7.4 (PBS), was injected 1.5  h later. Injection volumes did not exceed 1 ml/kg per animal. After the kainic acid injections, the rats were monitored continuously for 2 h for the development of epileptic symptoms.^48,86^

For MGV-1 post-treatment, rats first received a KA injection (9 mg/kg/rat) and then a single injection of 15 mg/kg MGV-1 2 h later, that is, after the typical period for full blown seizure activity. In a single experiment, each experimental group and the vehicle control group consisted of nine rats. Then, for 1 week, daily injections of MGV-1 were given, and the rats were tested daily for hyper reactivity in response to handling.^47^ Histological assays were applied 2 weeks after kainic acid injections.

#### Neurohistological assays of kainic acid-injected rats

For histological assays, rats that had developed seizures and had subsequently received treatment or control vehicle were sedated with ketamine (75 mg/kg) and xylazine (10 mg/kg) injections until no reflexes were seen and were then perfused with 4% of paraformaldehyde. Rats that had been pretreated with MGV-1 before kainic acid injections, and showed no seizures, were sedated and perfused in the same way. The brains were taken and then stored in paraformaldehyde 4% solution at 4 °C. After 48 h, the solution was changed to 20% sucrose solution at 4 °C. Brains of rats that were post treated with MGV-1 were prepared in the same way. After the brains had sunk they were embedded in optimal cutting temperature compound, frozen with liquid nitrogen, and stored at −70 °C. The brains were cut at −18 °C on a Cryostat Leica (Wetzlar, Germany) CM3050S microtome in 40 *μ*m sections from Bregma −2.8 to −5.8. The sections were kept in PBS without Ca^2+^ and Mg^2+^ with 0.5% sodium azide at 4 °C. Sections were incubated with primary antibodies against anti-NeuN to label neurons.^87^ The appropriate secondary antibodies were applied with VECTASTAIN Peroxidase ABC Kits (Vector Laboratories, Burlingame, CA, USA) based on avidin–biotin, according to the manufacturer’s instructions. The labeled, floating sections were mounted on microscope slides and coverslipped applying glycerol vinyl alcohol aqueous mounting solution. For observations and micrographs, a BH2 Olympus (Tokyo, Japan) upright microscope outfitted with a Nikon (Tokyo, Japan) DSFi1 digital camera was used.

#### The R6-2 transgenice mouse model for Huntington disease

Huntington disease in humans is a hereditary disease including neurodegeneration leading to degradation in motor performance, continual involuntary movements, tremors, cardiovascular and respiratory impairments, and finally leading to death of the patients having the disease.^88^ For this study, R6-2 mice (~120 CAG repeats B6CBA-Tg(HDexon1)62 Gpb/3J) mice were used as a transgenic mouse model for Huntington disease. For this purpose, mice obtained from the Jackson Laboratory were bred. In particular, males hemizygous for Tg(HDexon1)62 Gpb, and females from the wild-type background strain B6CBAF1/J, were bred according to the protocol of Jackson Laboratory.

#### Genotyping

With genotyping, we determined the presence of the Tg(HDexon1)62 Gpb gene in the parents and the offspring, according to standard methods as recommended by Jackson Laboratory and performed by Arnevet (Arnevet Preclinical Applications for Laboratory Animals, 2arnevet@gmail.com, Haifa, Israel). Tail tips were taken and kept at temperatures of ≤4 °C until the procedures for analysis performed at the same day. For DNA extraction, the tail tips were lysed in 75 *μ*l of 25 mM NaOH solution at 98 °C for 0.5  h. Then, the lysates were cooled to 4 °C and 75 *μ*l of 40 mM of Tris-HCl solution (pH 5.5) was added. To ensure high purity and quality of the DNA obtained we applied the QIAGEN DNA EXTRACTION KIT. PCR was performed using a DNA polymerase kit (DreamTaq PCR Master Mix (2X), of Thermo Scientific, Tamar Laboratory Supplies) and a thermal cycler (MJ Mini, Bio-Rad Laboratories, Rishon Le Zion, Israel).

To distinguish the mice carrying the Tg(HDexon1)62 Gpb mutation from the wild type, we used the following primers for the mutated alleles:

1. For the Tg(HDexon1)62Gpb mutation:

oIMR2093 – forward primer. Sequence: 5′-AAGCTAGCTGCAGTAACGCCATTT-3′.

oIMR2095 – reverse primer. Sequence: 5′-CTACAGCCCCTCTCCAAGGTTTATAG-3′.

The expected result is a 170-bp product (Jackson Laboratory).

1. For the number of CAG repeats in the Tg(HDexon1)62Gpb mutation:

oIMR6533 – forward primer. Sequence: 5′-GGCTGAGGAAGCTGAGGAG-3′.

TmoIMR1594 – reverse primer. Sequence: 5′-CCGCTCAGGTTCTGCTTTTA-3′.

Expected results are 566–596-bp products depending on the numbers of CAG repeats (120±5 CAG repeats).

The thermal protocol applied was:

– Initial denaturation: 2 min at 94 °C.
  - Denaturation: 30 s at 94 °C.
  - Annealing: 30 s at 65 °C.
  - Extension: 2 min at 72 °C.
– 35 cycles of:
  - Denaturation: 30 s at 94 °C.
  - Annealing: 30 s at 65 °C.
  - Extension: 2 min at 72 °C.
– Final extension: 10 min at 72 °C.

Gel electrophoresis was done with the aid of an E-Gel PowerBase apparatus (Invitrogen, Life Technologies, RHENIUM, Modi'in, Israel) using E-Gel 2% Double Comb gels applying the DNA Loading Dye #R0611 (Thermo Scientific, Waltham, MA, USA) and a 100-bp ladder. The gels were imaged under UV lighting using an LAS-3000 luminescent image analyzer (Fujifilm). CAG repeat evaluation was done against Invitrogen's Trackit ladder bands by AUTOCAD 2014 software (http://www.autodesk.com). For the male R6-2 breeders obtained from Jackson Laboratories, the numbers of CAG repeats were between 115 and 120, and this was true also for the two generations of their offspring we used to test the effects of 2-Cl-MGV-1 and 2-Cl-MGV-2, that is, within the 120±5 CAG range required for constancy in phenotype.

#### Drug efficacy assays

For tests of drug efficacy, male offspring hemizygous for Tg(HDexon1)62 Gpb were used to determine drug effect on lifespan, motor activity, and general well-being of the animals. Drug treatment for this study consisted of daily (5 continuous days per week) subcutaneous injections in the neck area of mice with 20 *μ*l of: the drug vehicle DMSO (vehicle control), 2-Cl-MGV-1(7.5 mg/kg) and 2-Cl-MGV-2 (7.5 mg/kg). Pilot studies also included application of saline (sham control), DMSO plus sesame oil (1 : 9), classic TSPO ligand PK 11195 (15 mg/kg), MGV-1 (15 mg/kg), and MGV-2 (15 mg/kg and 7.5 mg/kg). Quantities were used according to previous studies regarding TSPO ligands^48^ and adapted depending on the findings in the course of the pilot studies. The treatments were given until the animals died a natural death. Behavioral experiments included distance covered in an open field apparatus,^89^ freezing,^89^ and balance on a rotor-rod.^90^ Spontaneous tremor activity was evaluated using a Hamilton–Kinder sensor in a soundproof ventilated apparatus (Kinder Scientific). The assays for locomotor activity, tremors, and body weight were performed on a weekly basis, together with evaluation of the well-being of the mice. Finally, the day of death was noted. As lifespan data were most pronounced, these are presented in the present description of the results. No adverse effects of any of the drug treatments were noted, neither for specific tests, nor for general observations. Importantly, DMSO had no effect by itself as compared with naive mice or saline-injected mice with various behavioral assays.^20^

### Statistical analysis

Data are expressed as mean±S.E.M. or S.D., as stated for each experiment. The programs used for statistical analysis were Instat version-2 and graphpad prism (GraphPad Software, San Diego, CA, USA). When comparisons of more than two groups were needed then one-way analysis of variance (ANOVA) was performed with *post hocs*: Bonferroni's multiple comparisons test, Dunnett's multiple comparisons test, or Wilcoxon matched-pairs signed rank test, as appropriate. When required, the non-parametric Friedman test for repeated measures was used with Dunn's as a *post hoc*. When comparing only between two groups, Mann–Whitney's or Student's *t*-test for dependent or independent samples was performed as appropriate. The criterion for statistical significance was *P*<0.05.

## Abbreviations

2-Cl-MGV-1: 2-(2-chlorophenyl)quinazolin-4-yl dimethylcarbamate
2-Cl-MGV-2: 2-(2-chlorophenyl)quinazolin-4-yl ethyl(methyl)carbamate
2-Cl-MGV-3: 2-(2-chlorophenyl)quinazolin-4-yl diethylcarbamate
ΔΨm: mitochondrial membrane potential
ATCC: American Type Culture Collection
CA1: cornu ammonis area 1
CNS: central nervous system
GFAP: glial fibrillary acidic protein
JC-1: 5,5′,6,6′-tetrachloro-1,1′,3,3′-tetraethylbenzimidazolylcarbocyanine iodide a cationic carbocyanine dye that accumulates in mitochondria
Ki: inhibitory constant
LDH: lactate dehydrogenase
MGV: root name of compounds (Marek, Gavish, Veenman)
MGV-1: 2-phenylquinazolin-4-yl dimethylcarbamate
MGV-2: 2-phenylquinazolin-4-yl ethyl(methyl)carbamate
MGV-3: 2-phenylquinazolin-4-yl diethylcarbamate
NAO: fluorescent dye 10-*N*-nonyl-acridine orange
NeuN: antigen known as ‘neuronal nuclei’, also known as Rbfox3
NGF: nerve growth factor
NMDA receptors: *N*-methyl-D-aspartate receptors
PC12: cell line of pheochromocytoma origin with neuronal characteristics
PK 11195: the classical TSPO ligand: *N*-butan-2-yl-1-(2-chlorophenyl)-N-methylisoquinoline-3-carboxamide
R6-2: transgenic mouse model for Huntington disease; Strain name: B6CBA-Tg(HDexon1)62Gpb/3J
ROS: reactive oxygen species
TSPO: 18 kDa translocator protein
U118MG: glioblastoma cell line of glial origin.
